# Phytochemical profiling-guided green synthesis and characterisation of silver nanoparticles using *Catunaregam spinosa* for biological applications

**DOI:** 10.1371/journal.pone.0356646

**Published:** 2026-09-02

**Authors:** Rabina Baraili, Ishwor Pathak, Sugam Sharma, Dipak Raj Jaishi, Kanhaiya Lal Gupta, Khaga Raj Sharma

**Affiliations:** 1 Central Department of Chemistry, Tribhuvan University, Kirtipur, Kathmandu, Nepal; 2 Department of Chemistry, Amrit Campus, Tribhuvan University, Kathmandu, Nepal; 3 Department of Computer Engineering, Kathmandu Engineering College, Kathmandu, Nepal; 4 Department of Chemistry, Birendra Multiple Campus, Tribhuvan University, Bharatpur, Chitwan, Nepal; Al-Azhar University, EGYPT

## Abstract

In this study, an aqueous extract of *Catunaregam spinosa* (*C. spinosa*) leaves and roots was used to produce silver nanoparticles (AgNPs) in an environmentally friendly manner. Visual observation of the color change in the reacting solution and measurement of surface plasmon resonance by Ultraviolet-visible (UV-vis) spectroscopy at 414 nm for leaf-assisted silver nanoparticles (L-AgNPs) and 416 nm for root-assisted silver nanoparticles (R-AgNPs) were used to confirm nanoparticle (NPs) synthesis. Furthermore, NPs were examined by Fourier transform infrared spectroscopy (FTIR), X-ray diffraction (XRD), Field-emission scanning electron microscopy (FE-SEM), and Energy-dispersive X-ray analysis (EDX). FTIR spectra confirmed the utilization of various phytoconstituents as capping, reducing, and stabilizing agents during NPs formation. The average particle size of L-AgNPs in XRD was 11.57 ± 0.35 nm, while the average particle size of R-AgNPs was 10.05 ± 3.17 nm. The diameters of L-AgNPs (38.79 ± 0.62 nm) and R-AgNPs (39.93 ± 0.84 nm) were estimated using FE-SEM. The EDX investigation revealed that both AgNPs exhibited peaks at about 3 keV, as well as peaks for other elements such as O, N, Zn, C, and Cl. L-AgNPs showed substantial antioxidant activity, as determined by the enzyme marker 2,2-diphenyl-1-picrylhydrazyl (DPPH) with IC_50_ 106.10 ± 0.00 µg/mL. Furthermore, NPs exhibited strong antibacterial activity against human pathogenic bacterial strains. The toxicity of AgNPs was tested against brine shrimp nauplii, in which L-AgNPs (LC_50_ = 12.31 ± 11.93 µg/mL) were more toxic than R-AgNPs, indicating their potential for several biomedical applications.

## Introduction

Nanotechnology is the scientific field dedicated to the development and application of nanoscale materials. A notable increase in the number of products containing nanoworlds has occurred over the first three decades of the twenty-first century [1]. As technology has advanced significantly, it is now used across a wide range of sectors, including electronics, textiles, and most importantly, healthcare, where it is applied to biosensing, drug delivery, diagnostics, and treatment [2]. A nanoparticle (NPs) is a particle with dimensions less than 100 nm and greater than 1 nm, classified as ultrafine particles, which may or may not exhibit intense properties depending on size. There are numerous terms for nanotechnology, but “nanomaterial” and “NPs” are among the most commonly used. Although the two terms differ, they are occasionally used interchangeably [3]. There have been reports of metal nanoparticles (MNPs) (Ti, Au, Ag, Fe, Cu, and Zn) and their oxide counterparts (TiO_2_, Au_2_O_3_, Ag_2_O, FeO, CuO, and ZnO), with a wide range of medical applications, making them the preferred alternative [4–6].

Plant-assisted synthesis is the most successful bottom-up technique for synthesizing MNPs [7]. Plant extracts can create NPs by reducing metallic ions under appropriate conditions. The synthesis procedure is divided into three stages: (1) activation, during which metallic ions are reduced by phytoconstituents in various ways to prepare them for nucleation; (2) growth, during which NPs accumulate to form larger NPs; and (3) termination, which results in the production of NPs with a prescribed shape [8].

Bioactive substances, particularly flavonoids and phenols, serve as both stabilizing and reducing agents in the synthesis of MNPs. Interestingly, it adheres to the 12 principles of green chemistry, making it well-suited to a secure and sustainable future [9]. The production, properties, and applications of silver nanoparticles (AgNPs) can be altered by reaction variables, including light, pH, extraction concentration, and temperature. Various phytoconstituents diminish aggregation and precipitation by reducing van der Waals interactions, resulting in smaller particles, with plant extracts functioning as reducers and metal salts as precursors [10].

*Catunaregam spinosa* (Thunb.) Triveng (*C. spinosa*), a plant in the *Rubiaceae* family, is also known as mountain pomegranate. *C. spinosa* leaves have traditionally been used to treat gastrointestinal issues, skin disorders, tumors, piles, wound healing, snake bites, diarrhea, and dysentery. Pesticides and insect repellents are made from root extracts [11]. *C. spinosa* is regarded as the finest nausea treatment because it has no negative effects during emesis (Vamana Dravya) [12]. Pharmacological studies have shown that it contains phytochemicals such as phenols, flavonoids, alkaloids, terpenoids, and polysaccharides, which act as reducing agents to produce NPs with antioxidant properties and inhibit microbial proliferation. Hence, the primary goal of the research was to synthesize AgNPs from an aqueous extract of *C. spinosa* leaves and roots and to use principal component analysis (PCA) to correlate phytochemical content with antioxidant, antimicrobial, and toxicological activities.

## Materials and methods

### Chemicals

The entire experiment was done with deionized water. All chemicals and reagents utilized in this study were of high purity and analytical grade. Chemicals were purchased from Merck Life Science Private Limited in Germany, Thermo Fisher Scientific, HiMedia Laboratories Pvt. Ltd., Sisco Research Laboratories, and Srichem in India. The bacterial strains were from the American Type Culture Collection (ATCC).

### Plant sample collection and identification

**Fig 1:**
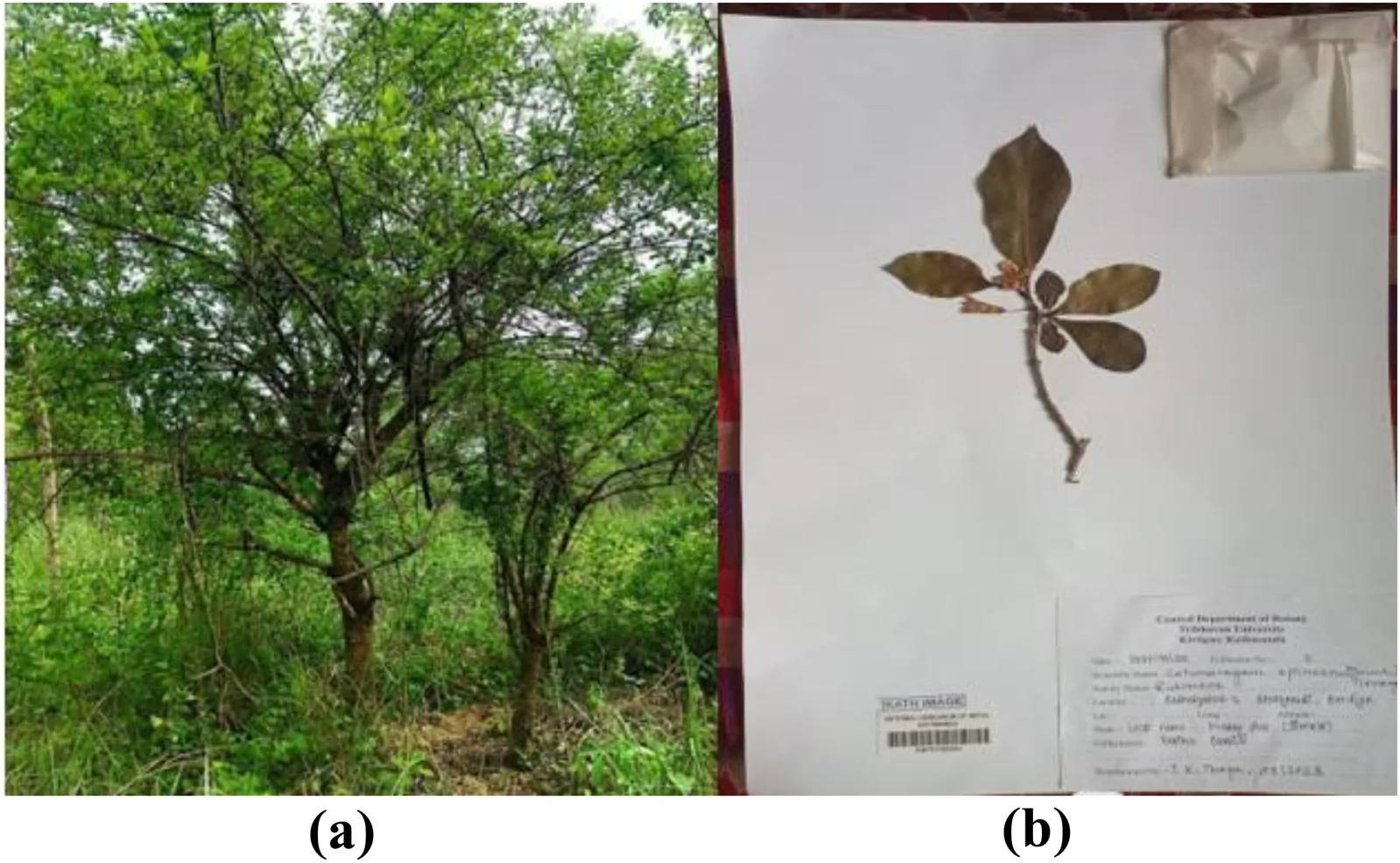
Fresh plant sample used in the study (a) *Catunaregam spinosa* (Thunb.) Triveng and (b) the herbarium of the plant sample.

*C. Spinosa* leaves and roots were collected from the Bardiya district in midwestern Nepal in June 2023. It lies 479 feet above sea level at latitude 28° 9′ 8″ N and longitude 81° 29′ 2″ E. The herbarium was prepared and identified (voucher code is RB-002 (KATH)). Fig 1(a) and (b) display pictures of *C. spinosa* and its herbarium.

### Determination of moisture content

Five hundred grams of fresh *C. spinosa* leaves and roots were washed with tap water to remove dust before being dried in the shade at room temperature for about 10 days. When the material was completely dry, it was ground into a powder using a pulverizer and stored in an airtight plastic bag for the following examination. The moisture content of the fresh plant samples was evaluated based on the prescribed method [13], using the equation Eq. (1).


$$\begin{array}{c} \begin{array}{c} \text{Moisture content }(\%)=\frac{\text{Fresh sample weight}-\text{Dry sample weight}}{\text{Fresh sample weight}}\times \text{100} \end{array} \end{array}$$ (1)

### Extraction

A hot magnetic stirrer was used to constantly agitate the mixture of five grams of finely powdered plant sample and 100 milliliters of distilled water while it was heated to 60 °C. The mixture was then filtered and stored at 4 °C for subsequent examination [14].

### Plant-assisted synthesis of silver nanoparticles

**Fig 2:**
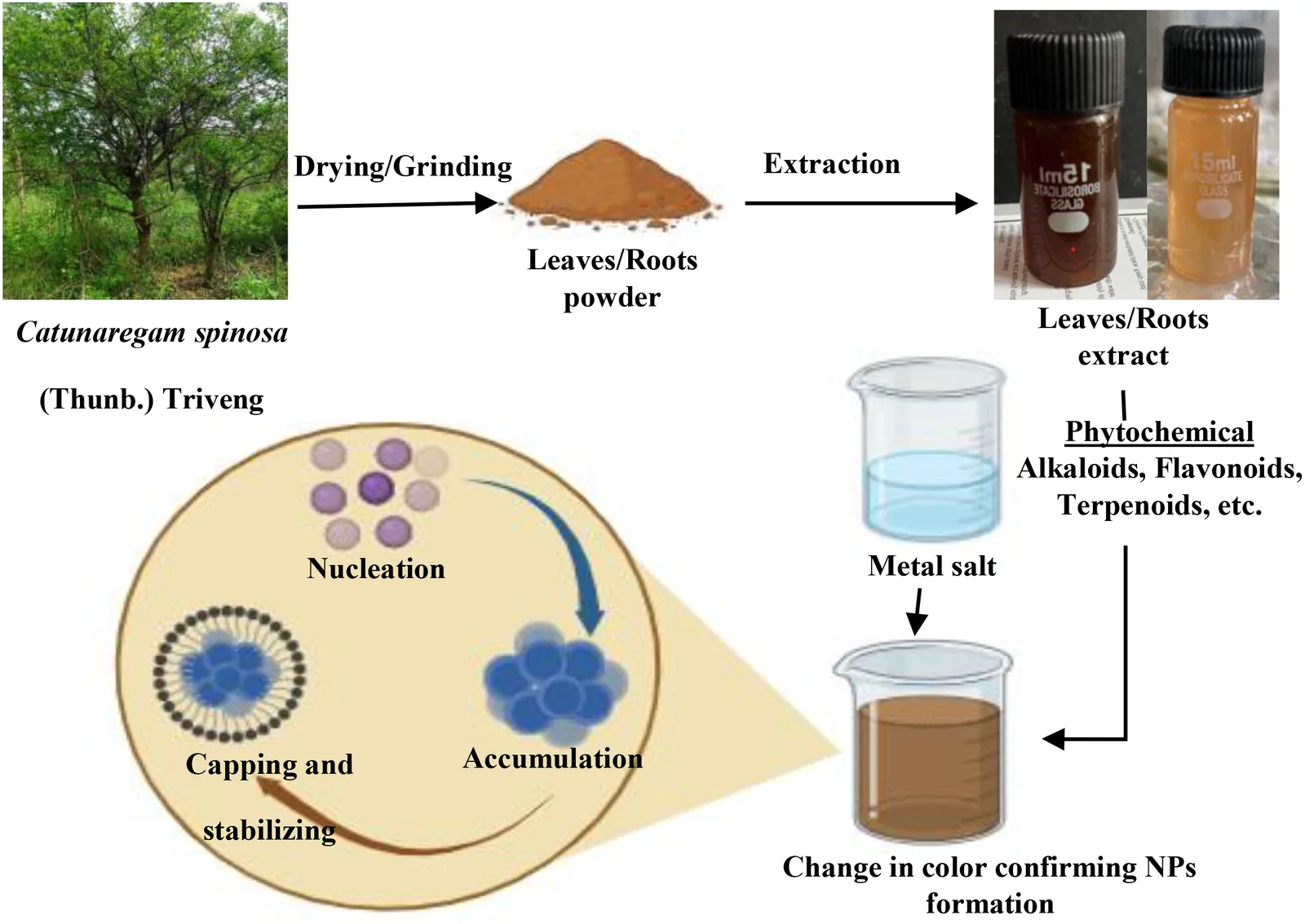
Plant-assisted synthesis of silver nanoparticles (AgNPs).

The AgNPs were successfully produced using a 20:1 volume proportion of AgNO_3_ (1 mM) to aqueous extract by following the standard methodology with minor changes [14,15]. AgNPs were produced at the optimal temperature of 60 °C with continuous stirring for 30 minutes. Initially, a rich reddish-brown hue in the reaction solution indicated the formation of AgNPs. The UV-vis spectra supported the reduction of Ag^+^. Finally, the AgNPs were extracted from the mixture using a high-speed centrifugation procedure that ran for 25 minutes at 9,000 revolutions per minute (rpm). After centrifugation, the AgNP pellets were washed with ethanol and distilled water, dried in a desiccator, and kept at 4 °C for further analysis. Fig 2 depicts the basic procedure for synthesizing AgNPs using plant extracts.

### Characterization of silver nanoparticles

The UV-vis spectra revealed an absorption peak at wavelengths ranging from 300 to 700 nm, confirming the formation of AgNPs. An organic functional group was identified on the surface of AgNPs using FTIR analysis. The spectra were scanned in the 400–4000 cm^−1^ range and processed using Origin 2019b (64-bit) software. The crystal structure of fine powder AgNPs was confirmed by XRD (Bruker D2 Phaser, NAST, Nepal) at a wavelength of 1.540 Å and a voltage of 30 kV. Working conditions generally required 2θ scanning, which was between 10° and 20°, and the captured data was evaluated using Origin 2019b (64-bit) software. The crystallite size was estimated using Eq. (2). The sample’s size, shape, composition, crystallography, and other physical and chemical properties were investigated and magnified using a FE-SEM (SU-70 instrument from Korea). The sizes of the AgNPs were determined using ImageJ software. Using EDX analysis, distinct elemental compositions within the NPs were discovered.


$$\begin{array}{c} \begin{array}{c} \text{ D }=\frac{k\lambda }{\beta cos\theta } \end{array} \end{array}$$ (2)

Where λ is the x-ray radiation wavelength, k is the dimensionless factor (~1), D is the crystallite grain size, β is the full width at half maximum (FWHM) in radians, and θ is half of the 2θ value of the chosen peak in radians (Bragg’s angle).

### Qualitative phytochemical analysis

Phytochemicals present in aqueous crude extract, such as flavonoids, alkaloids, tannins, terpenoids, anthraquinones, phenols, saponins, steroids, glycosides, and carbohydrates, were estimated by applying standard procedures qualitatively [16,17].

### Estimation of total phenolic content (TPC), total tannin content (TTC), and total flavonoid content (TFC)

The Folin-Ciocalteu reagent (FCR) was used to determine TPC and TTC in extracts, with slight modifications [18,19]. For TPC, 20 µL of each plant extract solution was added to 100 µL of diluted FCR at different dosages and 80 µL of a 1 M sodium carbonate (Na_2_CO_3_) solution. For TTC, 10 µL of extract was combined with 70 µL of distilled water, 50 µL of FCR, and 70 µL of 1 M Na_2_CO_3_. The absorbance of each solution was measured at 765 nm for TPC and 725 nm for TTC. The TFC was measured using aluminum chloride (AlCl_3_) colorimetry [19]. For TFC, 100 µL of distilled water, 60 µL of ethanol, 10 µL of potassium acetate, and 10 µL of a 10% AlCl_3_ solution were combined with 20 µL of each extract. The absorbance was measured at 415 nm. TPC, TTC, and TFC were calculated and expressed as milligrams of Gallic acid, Tannic acid, and Quercetin equivalents per gram of dry material, as shown in Eq. (3).


$$\begin{array}{c} \begin{array}{c} \text{C}=\frac{\text{cV}}{\text{m}} \end{array} \end{array}$$ (3)

where m is the dry mass of the plant extract in grams, V is the volume of the plant extract solution in milliliters, C is TPC, TTC, or TFC, and c is the concentration of Gallic acid, Tannic acid, or Quercetin (mg/mL), which corresponds to the plant extract’s absorbance in the relevant calibration curves.

### Evaluation of antioxidant potential

The antioxidant activity of the plant extract and NPs was assessed using the 2,2-diphenyl-1-picrylhydrazyl (DPPH) radical scavenging ability [20,21]. The aqueous extract of *C. spinosa* leaves and roots (100 μL) was treated with a methanolic solution of DPPH (100 μL, 0.1 mM) at different concentrations (10, 25, 50, 75, 100, 250, and 500 μg/mL) and NPs (15.625 μg/mL to 250 μg/mL). The mixture was left in the dark for 30 minutes before the absorbance was measured at 517 nm. Each test was performed in triplicate, with Quercetin as the reference standard (0.625 μg/mL to 20 μg/mL). To figure out the percentage inhibition, Eq. (4) was used.


$$\begin{array}{c} \begin{array}{c} \text{Percentage scavenging }(\%)=\frac{\left(\text{Acontrol}-\text{Asample}\right)}{\text{A control }}\times \text{100 } \end{array} \end{array}$$ (4)

Where, A _control_ = absorbance of control, A _sample_ = absorbance of sample

### Evaluation of antibacterial activity

The agar diffusion well method was employed to examine antibacterial activity in line with a predetermined approach [22]. Four distinct bacterial pathogens were utilized to assess the antibacterial efficacy of the samples. Three Gram-negative bacteria are *Shigella sonnei* (ATCC 25931), *Klebsiella pneumoniae* (ATCC 700603), and *Escherichia coli* (ATCC 25912), with one Gram-positive bacterium, *Staphylococcus aureus* (ATCC 43300). The pathogens were injected into Mueller-Hinton Broth (MHB) and incubated at 37 °C. A final inoculum of 1.5 × 10^8^ CFU/mL was obtained after correcting the turbidity to the standard 0.5 McFarland. In each experiment set, 25 µL of test sample (50 mg/mL), 100% distilled water (negative control), and 1 mg/mL of neomycin (positive control) were added to individual wells. The content was incubated for 24 hours at 37 °C after a 15-minute diffusion period at room temperature. The ZOI (mm) around the well was visible on the plates after incubation.

### Determination of minimum inhibitory concentration (MIC) and minimum bactericidal concentration (MBC)

The resazurin microdilution assay was performed to determine the minimum inhibitory concentration (MIC) based on the standard procedure [23]. MIC is the lowest concentration of a drug that inhibits an organism’s visible growth after an overnight incubation period (extended for anaerobes that require longer incubation for growth). In contrast, minimum bactericidal concentration (MBC) is the minimum concentration of an antibacterial agent required to kill bacteria, rather than just inhibit growth [24]. For MIC, the 0.5 McFarland turbidity culture was diluted 1:100 in Mueller-Hinton broth (MHB) to produce the bacterial inoculum, which had a final concentration of 10^6^ CFU/mL. Finally, each well received 5 µL of bacterial injection. The positive control was neomycin, a well-known antibiotic. The microtiter plate was sealed with a sterile cover before incubating for 24 hours at 37 °C. Plates were then filled with 0.003% resazurin and incubated at 37 °C for four hours. Wells with no bacterial growth remained blue, but those with bacterial growth turned pink. For MBC, the contents of the well were incubated again at 37 °C for 24 hours after being streaked onto nutrient agar plates. MIC and MBC of the NPs were determined only for one gram-positive and one gram-negative bacterium against which NPs exhibited the greatest antibacterial effect in the agar diffusion well method.

### Toxicity analysis

**Fig 3:**
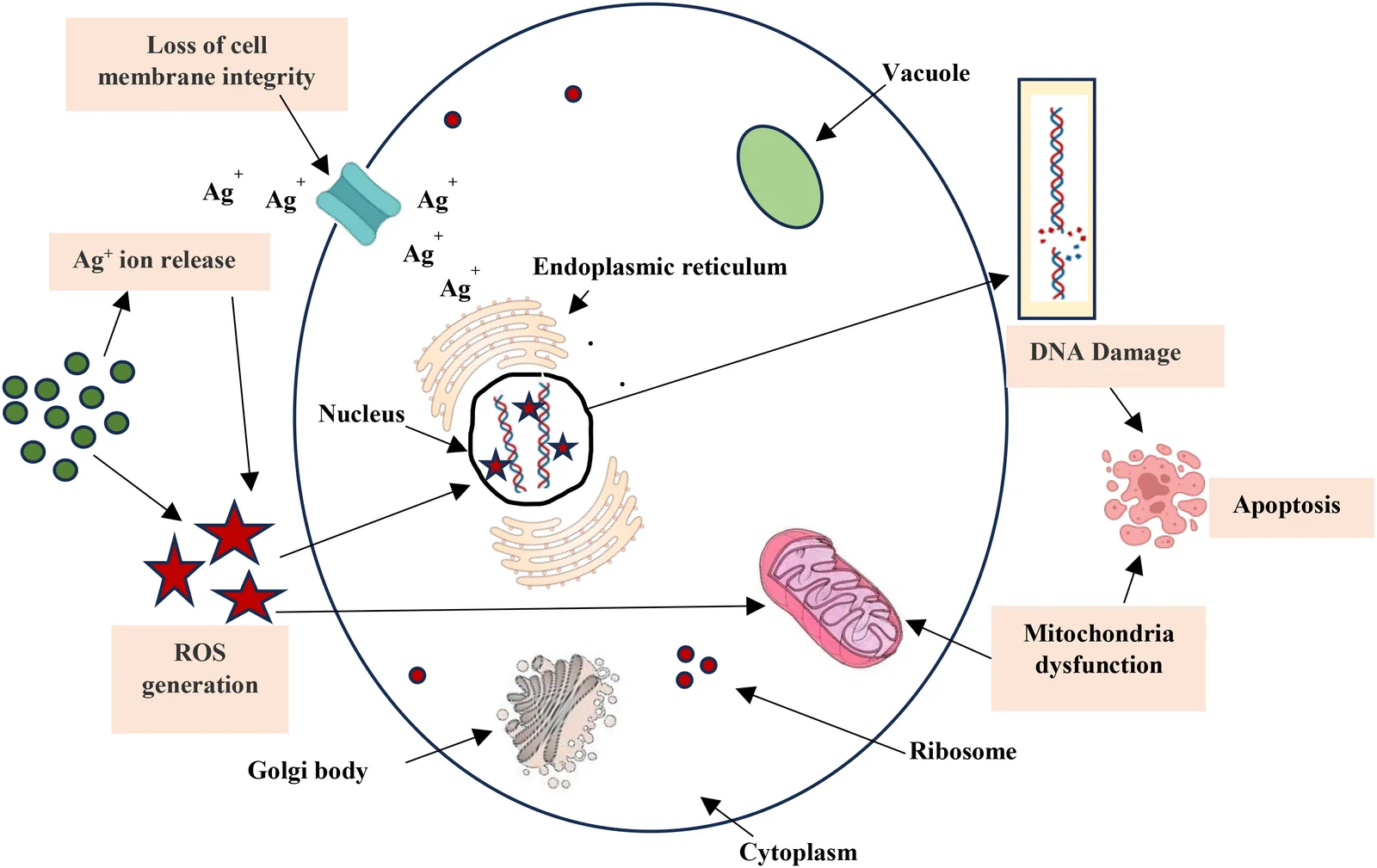
Basic mechanism showing the toxic activity of silver nanoparticles (AgNPs).


A brine shrimp mortality assay was employed to assess toxicity [25]. One liter of double-distilled water was combined with 40.567 grams of salt to create artificial seawater. The pH was maintained between 8 and 8.5 by using 1N NaOH. Under the proper conditions, brine shrimp cysts were hatched. Ten newly hatched nauplii were exposed to a 5 mL test solution with 4 mL of saline water and 1 mL of test sample concentrations (1000, 500, 250, 125, 100, and 10 µg/mL), then incubated for the entire day. The dose optimized for the study was based on available literature [26]. After 24 hours, the number of viable nauplii in each test tube was counted, and percentage mortality was calculated using Eq. (5). Fig 3 depicts the basic mechanism of AgNPs toxicity.

$$\begin{array}{c} \begin{array}{c} \text{M }(\%\text{ vs}.\text{ control})=\frac{\;(\text{Nc}-\text{Nt})\;}{\text{Nc}}\times \text{ 100} \end{array} \end{array}$$ (5)

Where Nc is the number of live nauplii in the control after 24 hours, Nt is the number of living nauplii after 24 hours, and M is the mortality rate.

### Statistical analysis

The data were presented as the mean ± SD of triplicate tests. The calibration curves for TPC, TTC, and TFC were generated using Microsoft Excel 2019. In the DPPH and brine shrimp assays, the IC_50_ and LC_50_ values were calculated with GraphPad Prism 8.0.2 (GraphPad Software Inc., Boston, Massachusetts, USA) and Excel, which also served to create the relevant graphics. The FTIR, XRD, and UV-visible spectra were all plotted using OriginPro 2019b (OriginLab Corporation, Northampton, Massachusetts, USA). ImageJ 1.54g (National Institutes of Health, Bethesda, Maryland, USA) was used to analyze SEM images.

## Results

### Characterization of silver nanoparticles

#### Ultraviolet-visible (UV-vis) spectroscopy.

**Fig 4:**
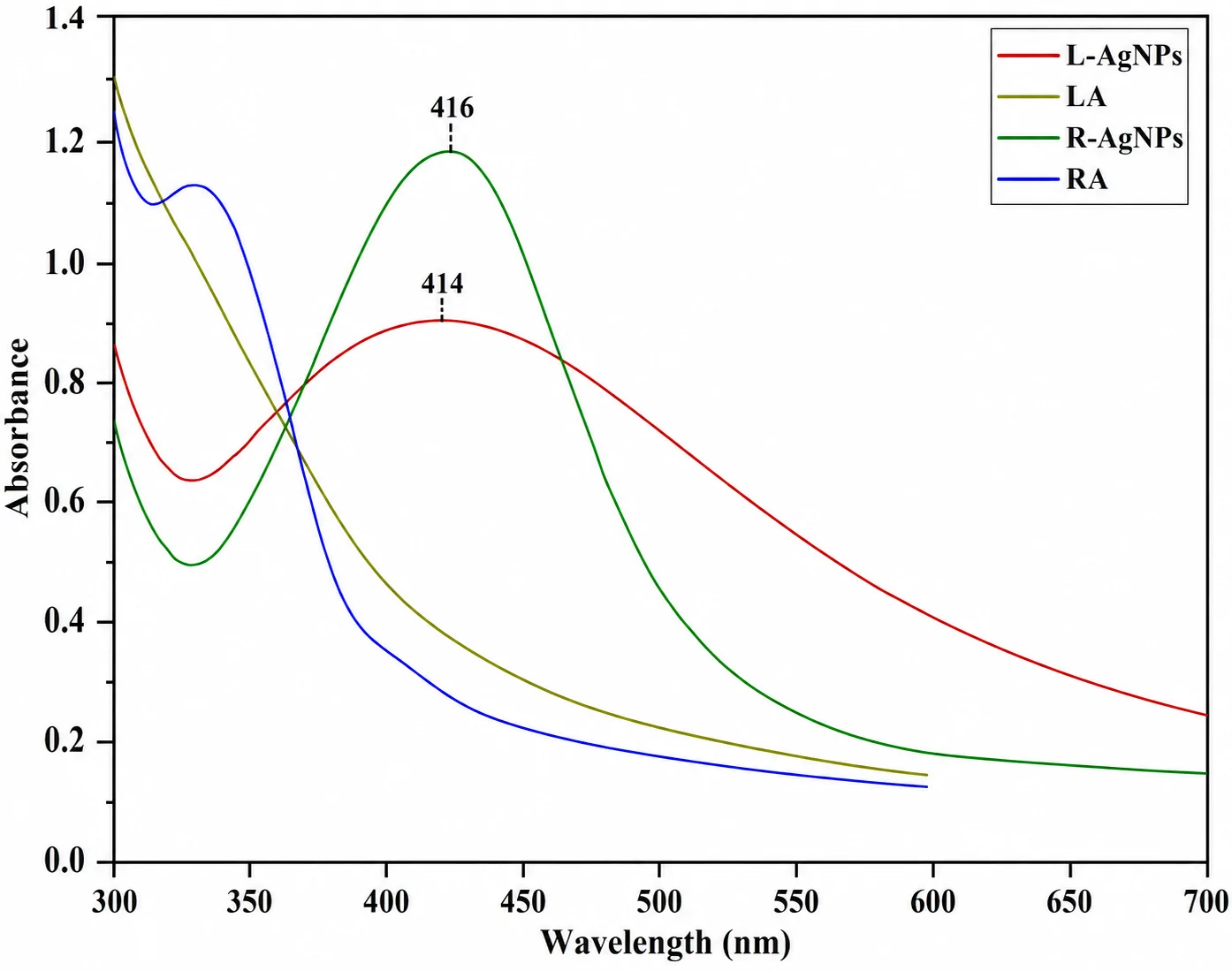
UV-vis spectrum of aqueous extracts and their respective nanoparticles.

The highest absorption peaks were observed at 414 nm for leaf-assisted silver nanoparticles (L-AgNPs) and 416 nm for root-assisted silver nanoparticles (R-AgNPs). It was found that the optimal time to obtain stable AgNPs was approximately 24 hours of incubation. Larger AgNPs display broad peaks and a redshift (absorb at longer wavelengths), while smaller, spherical AgNPs absorb around 400 nm. Additionally, the expansion and weakening of peaks, along with the appearance of secondary peaks at higher wavelengths due to particle agglomeration, suggest the stability of AgNPs [27]. Fig 4 shows the UV-vis spectrum of aqueous extracts and their respective NPs.

#### Fourier transform infrared spectroscopy (FTIR).

**Fig 5:**
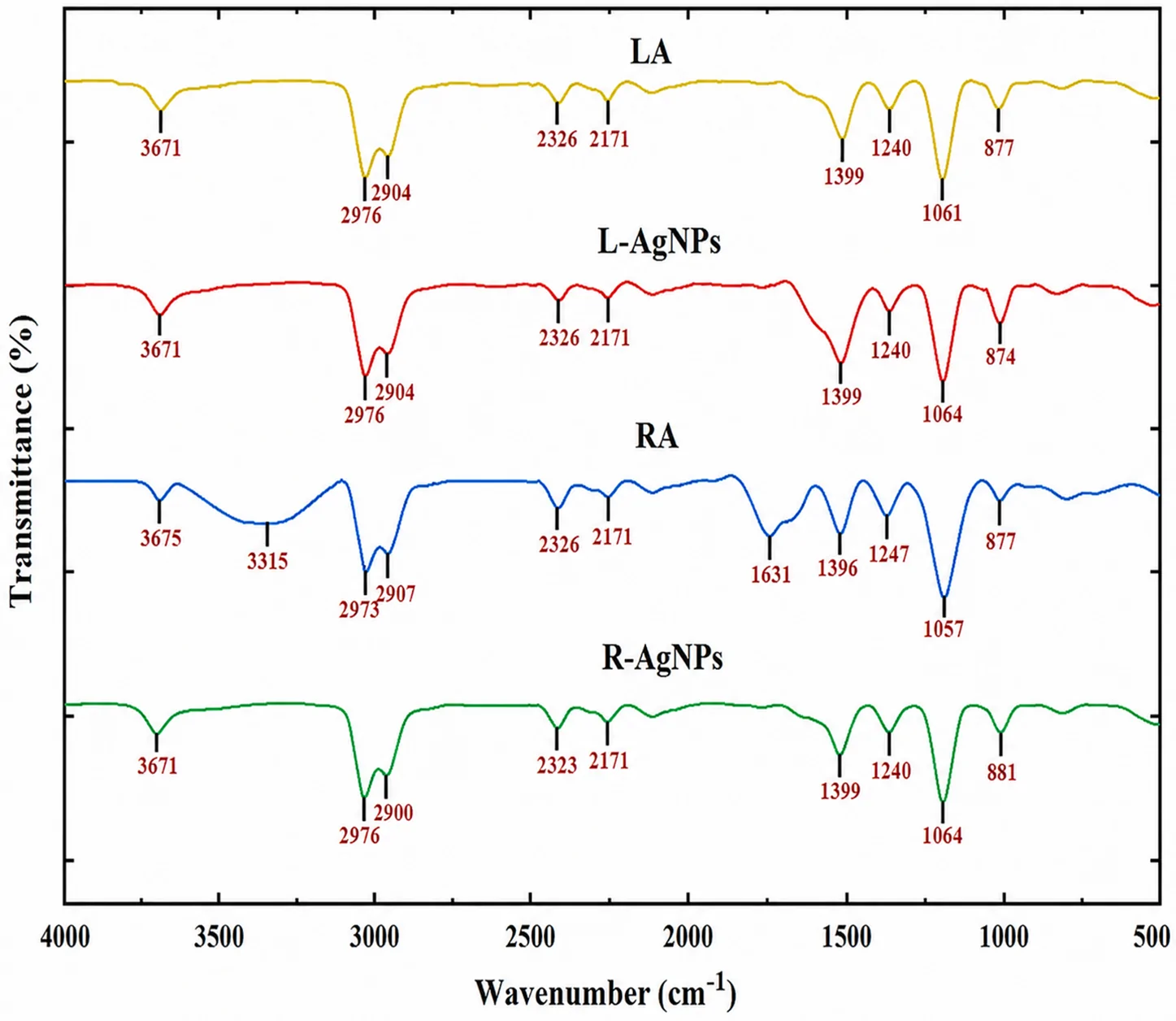
FTIR spectrum of aqueous extracts and their respective nanoparticles.

The amount of hydrogen bonding, biomolecular binding information, particularly in the formation of AgNPs, and the identification of functional groups all show changes in peak location between FTIR spectra of plant extracts and those of AgNPs. Fig 5 shows the FTIR spectra of the aqueous extracts and their corresponding NPs. The spectral patterns of the aqueous leaf extract (LA) include peaks at 877, 1061, 1240, 1399, 2171, 2326, 2904, 2976, and 3671 cm^-1^. Meanwhile, the AgNPs display nearly the same peaks, with slight reductions in wavenumber. Peaks in the 874–881 cm^-1^ range suggest the presence of C-S or Si-O bonds, while bands from 1061 to 1064 cm^-1^ may result from C-O stretching in alcohols, ethers, or esters, indicating flavonoids adsorbed on the MNPs surface [28]. The peaks at 1240 cm^-1^ and 1399 cm^-1^ correspond to C-O or C-N stretching vibrations and the symmetric stretching of carboxylate (-COO^-^) groups or C-H bending, respectively, indicating the presence of oxygen-containing functional groups involved in surface capping or material stabilization. The peaks at 2171–2326 cm^-1^ could be interference or noise, while the peaks at 2904–2976 cm^-1^ indicate aliphatic C-H stretching (-CH_2_/-CH_3_ groups), and the band at 3671 cm^-1^ corresponds to free O-H stretching, indicating the presence of non-hydrogen-bonded hydroxyl groups. Following the plant-assisted synthesis of NPs, the aqueous root extract (RA) showed a spectrum with peaks at 877, 1057, 1247, 1396, 1631, 2171, 2326, 2907, 2973, 3315, and 3675 cm^-1^. The NPs, in turn, exhibited maxima at 881, 1064, 1240, 1399, 2171, 2323, 2900, 2976, and 3671 cm^-1^. Unlike the RA, its NPs lack -OH bending or stretching at 1631 cm^-1^ and 3315 cm^-1^.

#### X-ray diffraction (XRD).

**Fig 6:**
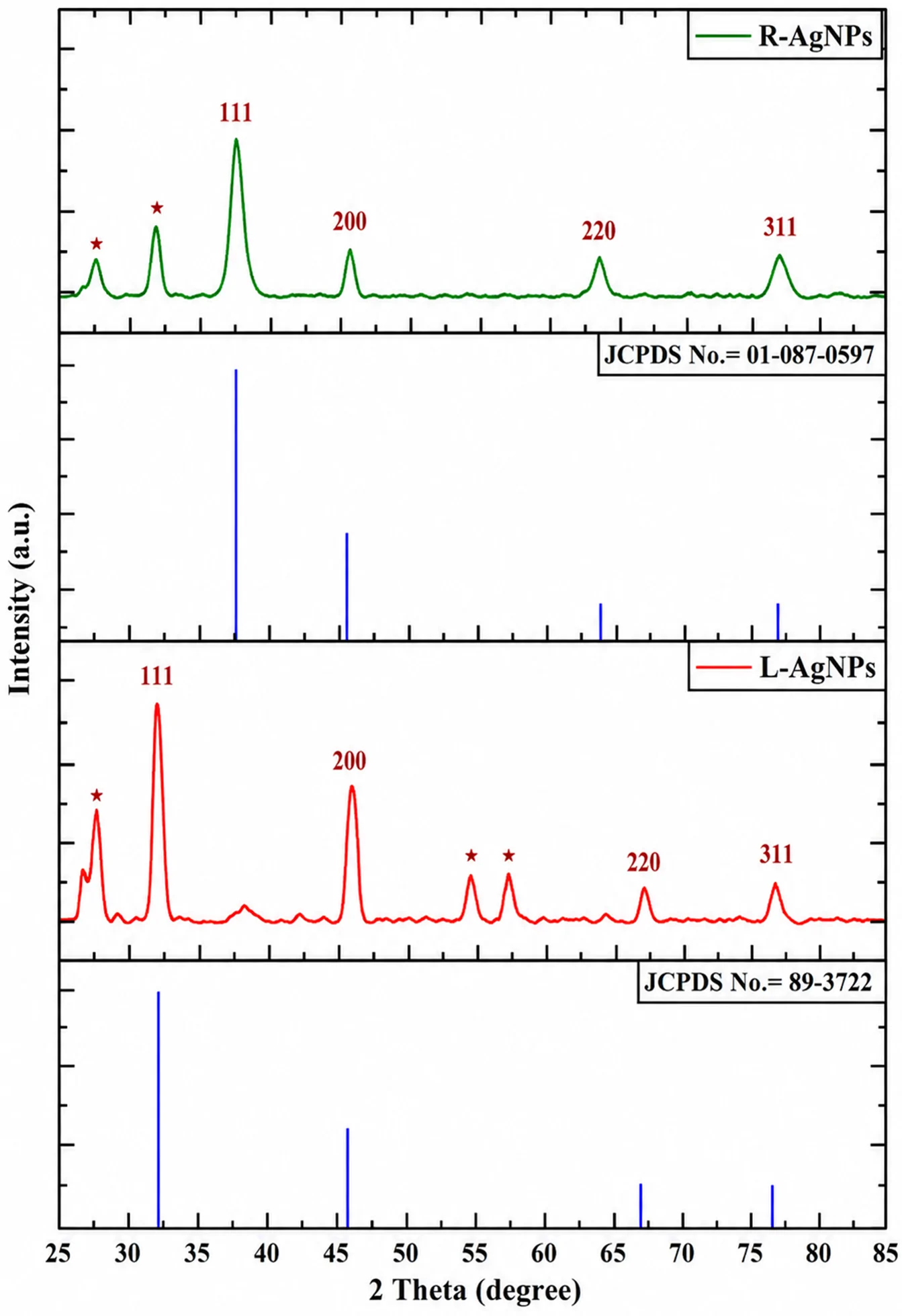
XRD pattern of leaf-assisted silver nanoparticles (L-AgNPs) and root-assisted silver nanoparticles (R-AgNPs).

As shown in Fig 6, the results clearly show the formation of crystalline AgNPs. These findings align with data from the Joint Committee on Powder Diffraction Standards (JCPDS), which helps to identify phases in XRD patterns [29]. The spectrum of L-AgNPs revealed four prominent peaks: (111), (200), (220), and (311). The Bragg’s reflection (2θ) values were 32.23°, 46.25°, 67.53°, and 76.84°, corresponding to JCPDS card no. 89–3722. Similarly, the Bragg’s reflection (2θ) values for R-AgNPs were 37.91°, 45.99°, 64.22°, and 77.10°, with four significant peaks matching (111), (200), (220), and (311) crystalline planes, linked to JCPDS card no. 01-087-0597. The width of each diffraction peak was estimated using the Debye-Scherrer formula after fitting the peaks with a Gaussian function to determine the average crystallite size of bio-reduced L-AgNPs and R-AgNPs, and the values were 11.57 ± 0.35 nm and 10.05 ± 3.17 nm, respectively.

#### Field emission scanning electron microscopy (FE-SEM).

**Fig 7:**
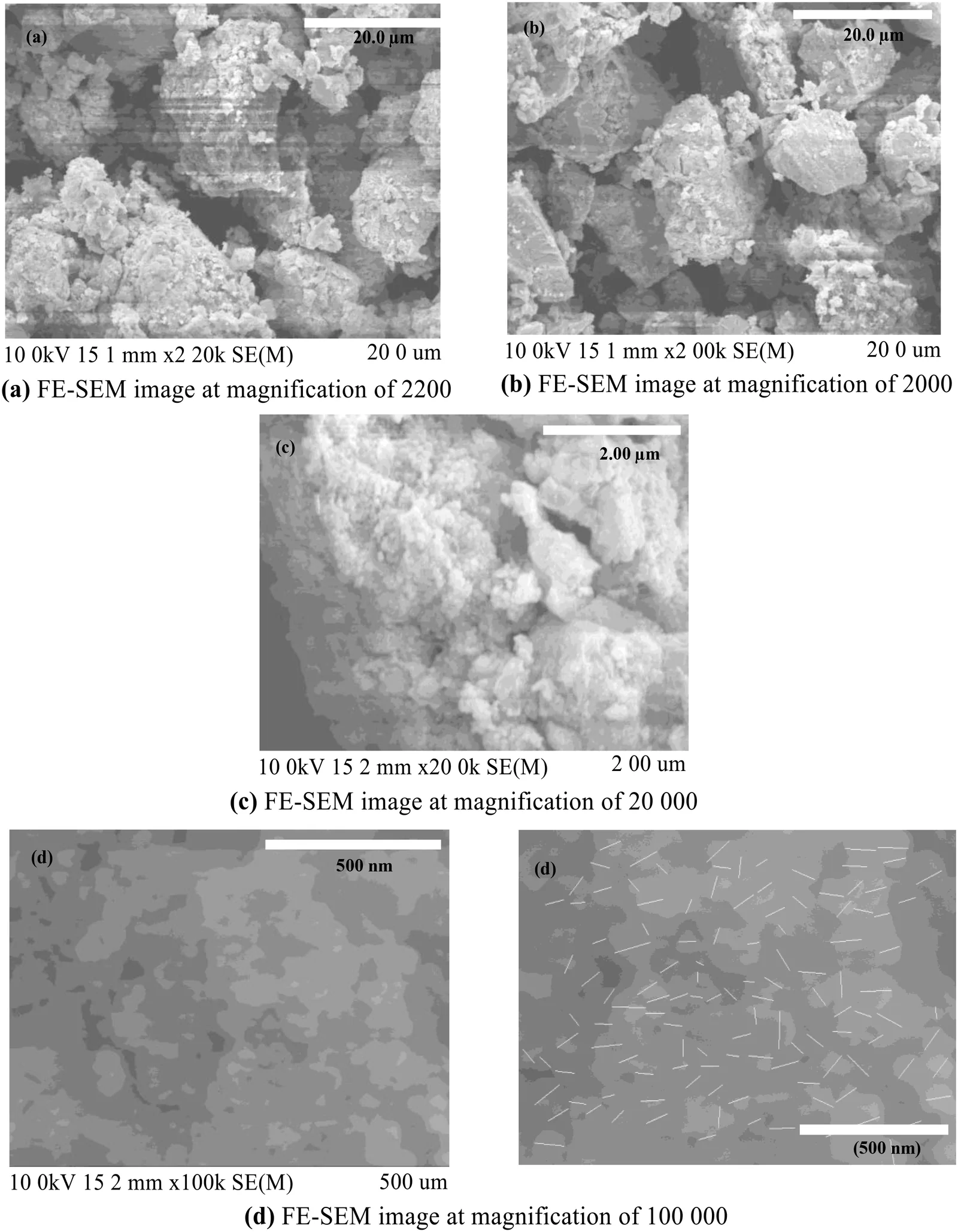
FE-SEM images of leaf-assisted silver nanoparticles (L-AgNPs) at different magnifications (a) FE-SEM image at the magnification of 2000, (b) FE-SEM image at the magnification of 2,200, (c) FE-SEM image at the magnification of 20,000, and (d) FE-SEM image at the magnification of 100,000.

**Fig 8:**
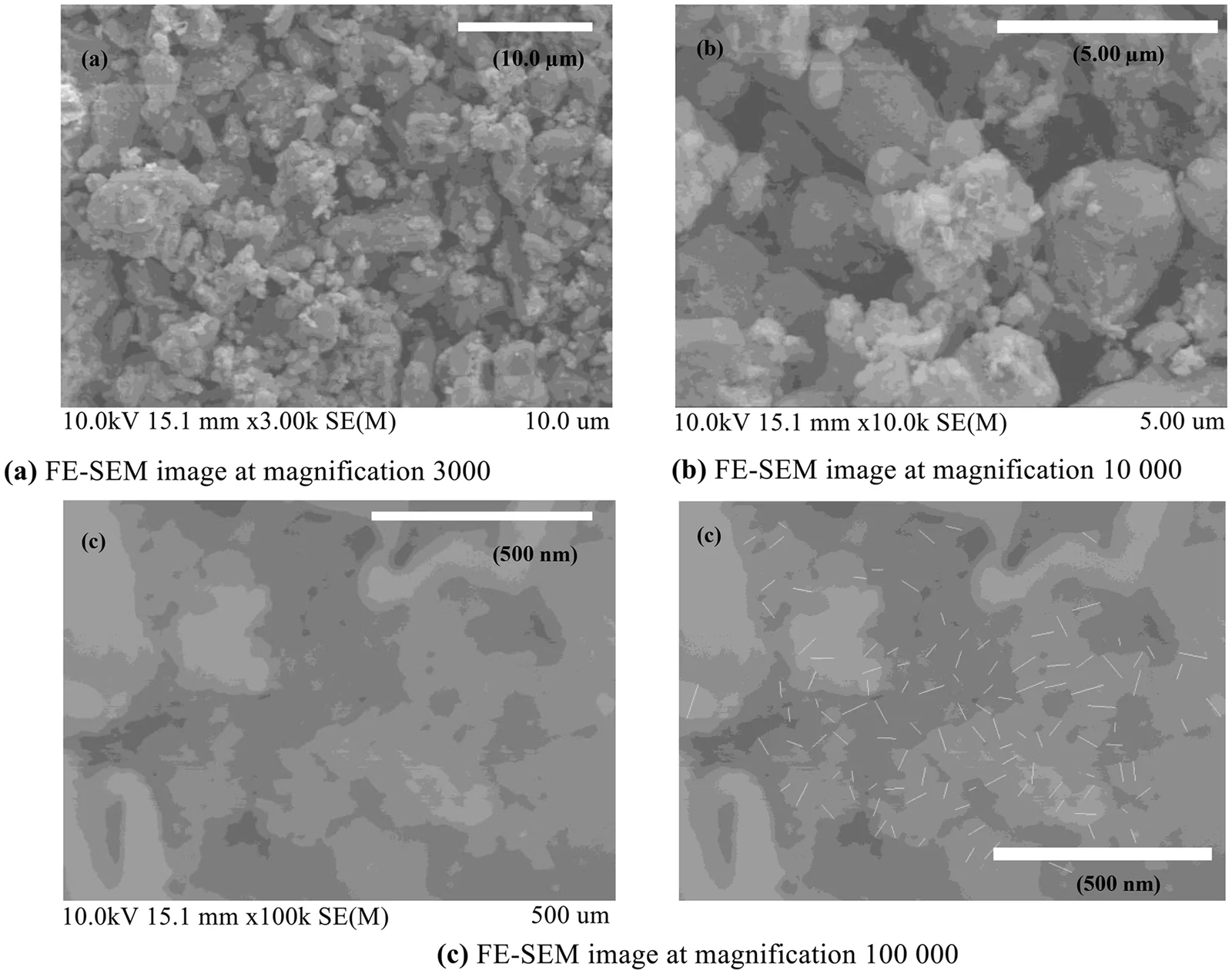
FE-SEM images of root-assisted silver nanoparticles (R-AgNPs) at different magnifications (a) FE-SEM image at the magnification of 3000, (b) FE-SEM image at the magnification of 10,000, and (c) FE-SEM image at the magnification of 100,000.

**Fig 9:**
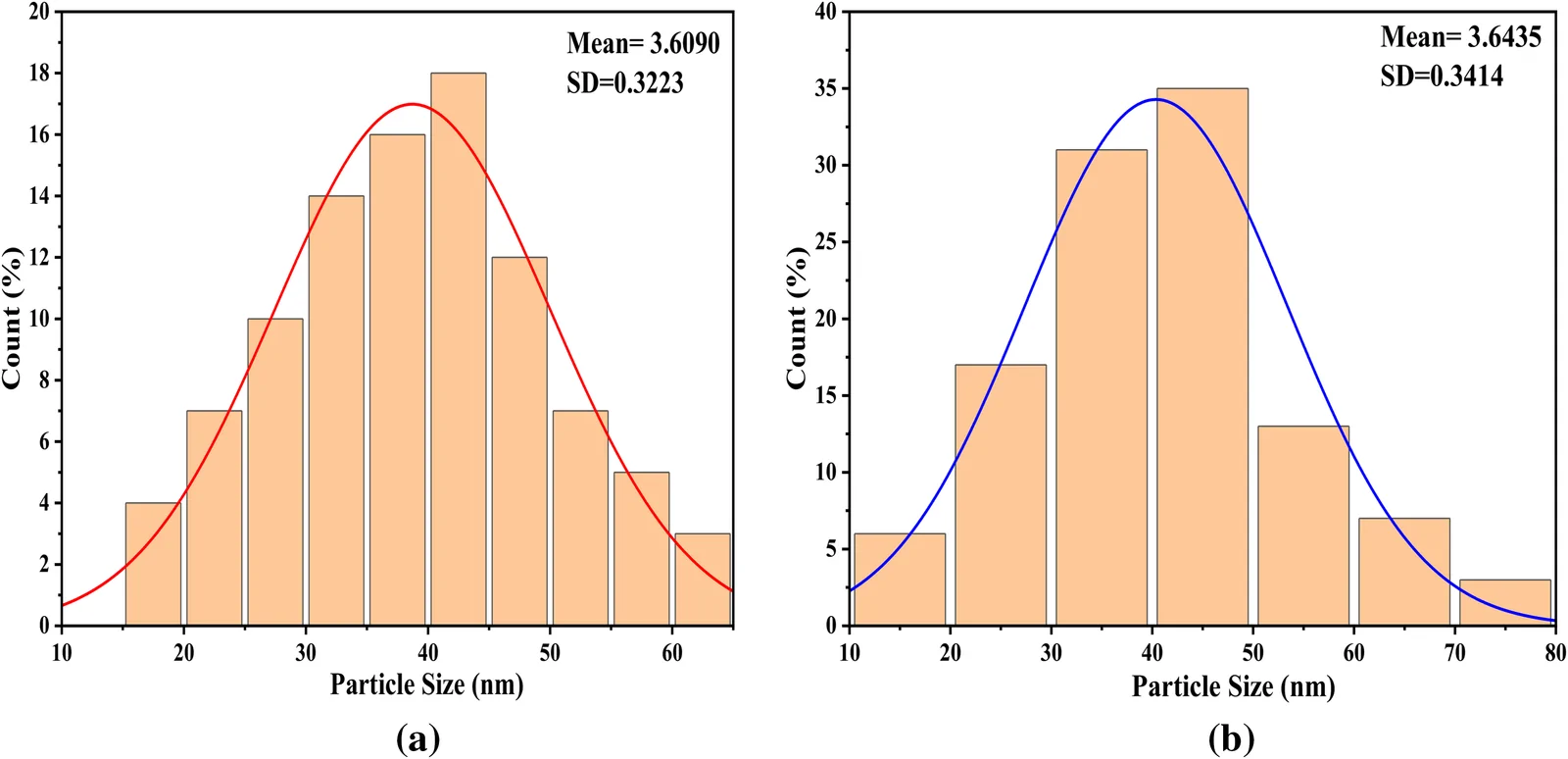
SEM images showing average particle size distribution through a histogram for (a) leaf-assisted silver nanoparticles (L-AgNPs) and (b) root-assisted silver nanoparticles (R-AgNPs).

FE-SEM showed that the *C. spinosa* extracts produced high-density NPs. The bio-organic capping and stabilizing molecules attached to the NPs interact through electrostatic and hydrogen bonds during the plant-assisted synthesis of AgNPs [30]. The diameters of L-AgNPs and R-AgNPs were measured at 38.79 ± 0.62 nm and 39.93 ± 0.84 nm, respectively. Fig 7(a)–(d) show FE-SEM images of L-AgNPs at different magnifications (a) = 2,000, (b) = 2,200, (c) = 20,000, and (d) = 100,000. Fig 8(a)–(c) show FE-SEM images of R-AgNPs at (a) = 3,000, (b) = 10,000, and (c) = 100,000. Fig 9(a) and 9(b) illustrate SEM images of average particle size through histograms for L-AgNPs and R-AgNPs.

#### Energy-dispersive X-ray analysis (EDX).

**Fig 10:**
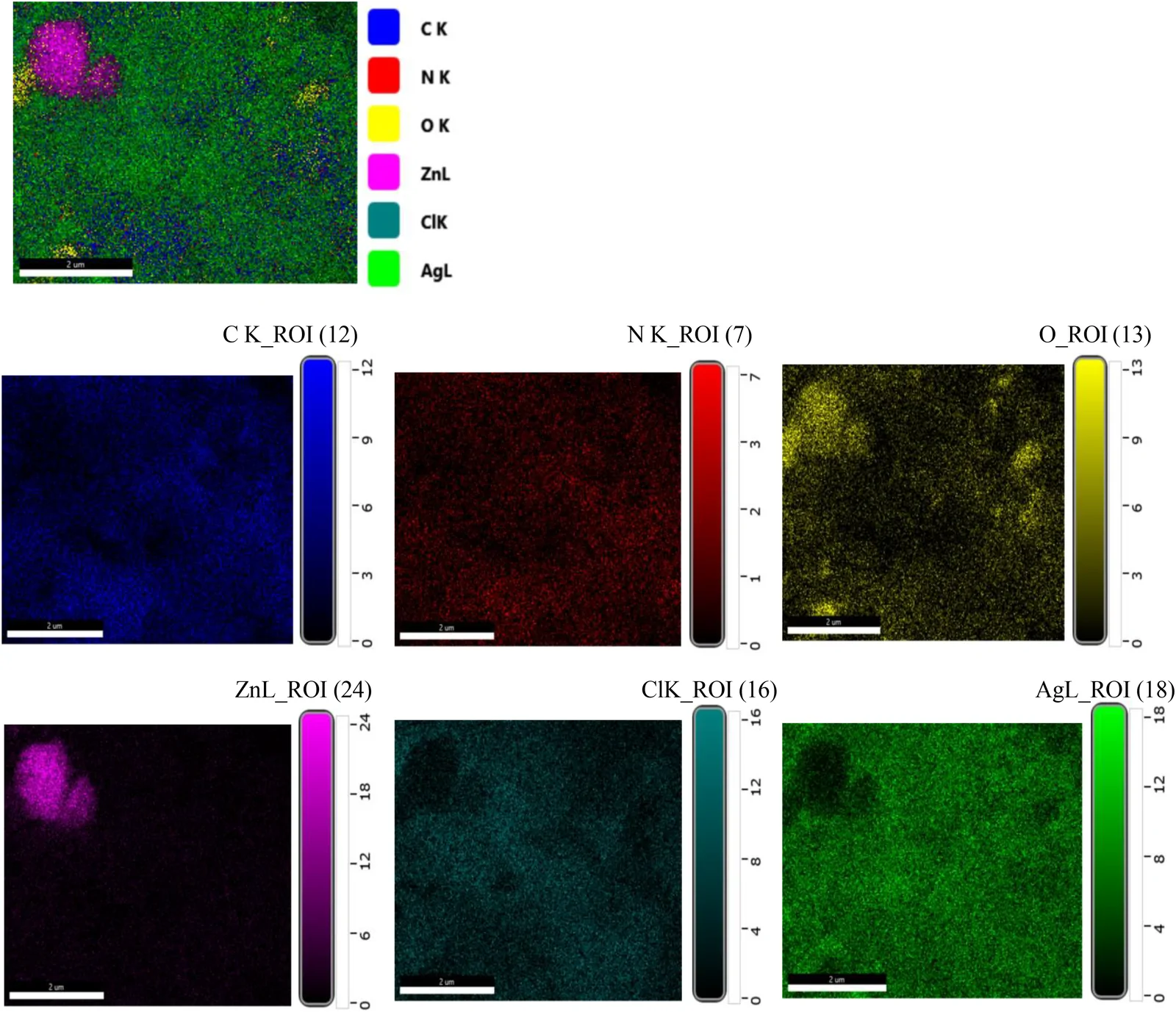
EDX spectrum of leaf-assisted silver nanoparticles (L-AgNPs) with total elemental mapping and individual color distribution.

**Fig 11:**
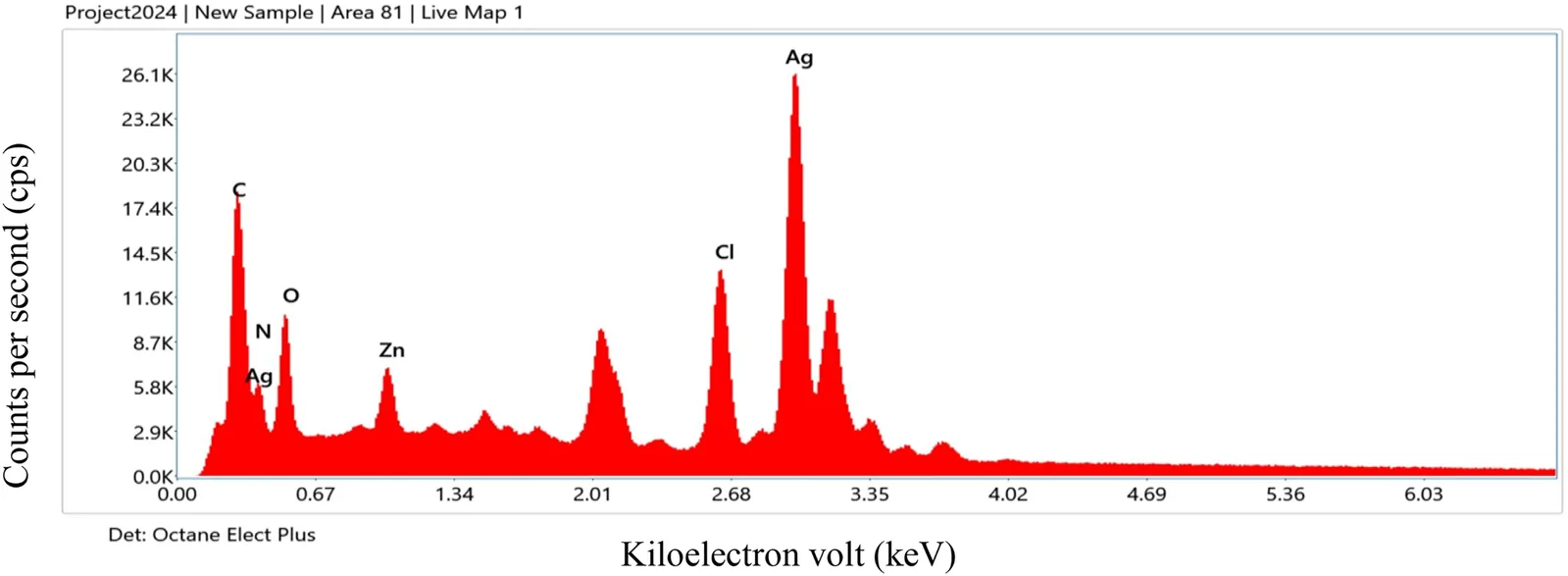
EDX spectrum of leaf-assisted silver nanoparticles (L-AgNPs).

**Fig 12:**
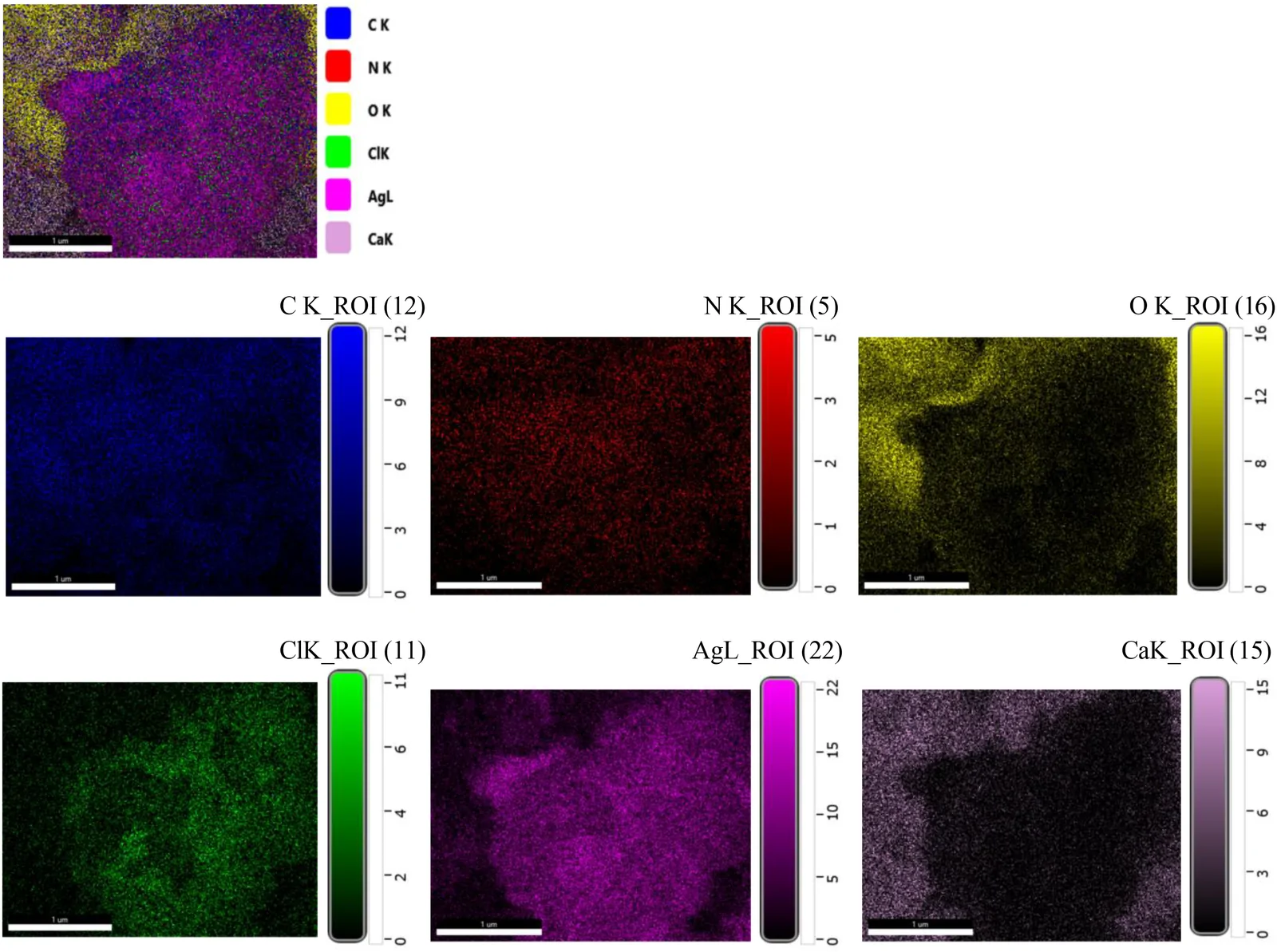
EDX spectrum of root-assisted silver nanoparticles (R-AgNPs) with total elemental mapping and individual color distribution.

**Fig 13:**
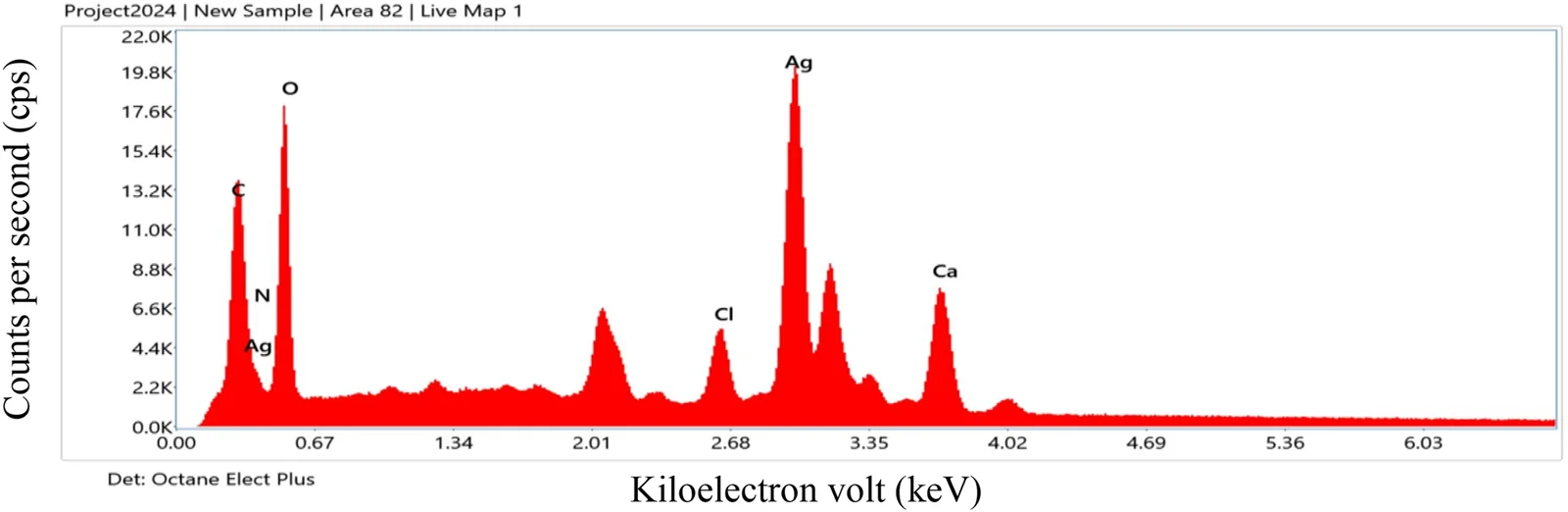
EDX spectrum of root-assisted silver nanoparticles (R-AgNPs).

The EDX images displayed the color map and analysis confirming the presence of silver. The analysis showed peaks around 3 keV for each AgNP, with other elements such as O, N, Zn, C, and Cl. Figs 10 and 11 show the EDX spectrum of L-AgNPs, including total elemental mapping and individual color distributions. Figs 12 and 13 show the EDX spectrum of R-AgNPs, including total elemental mapping and individual color distributions.

### Moisture content

Following 10 days of drying at room temperature, the fresh weights of the plant samples reduced considerably. The leaves weighed 500 g fresh but only 373.15 g after shade-drying. The moisture content was estimated to be 25.37% by subtracting the dried weight from the fresh weight. In the same way, the root weighed 500 g fresh but dropped to 443.35 g after shade-drying. The moisture content was calculated at 11.33%, indicating that water comprised a significant portion of the plant tissue.

### Phytochemical analysis

The study found that the most prevalent phytochemicals in this species were alkaloids, phenols, flavonoids, tannins, and carbohydrates; none of the extracts included terpenoids, steroids, or anthraquinones.

### Total phenolic content (TPC), total tannin content (TTC), and total flavonoid content (TFC)

**Fig 14:**
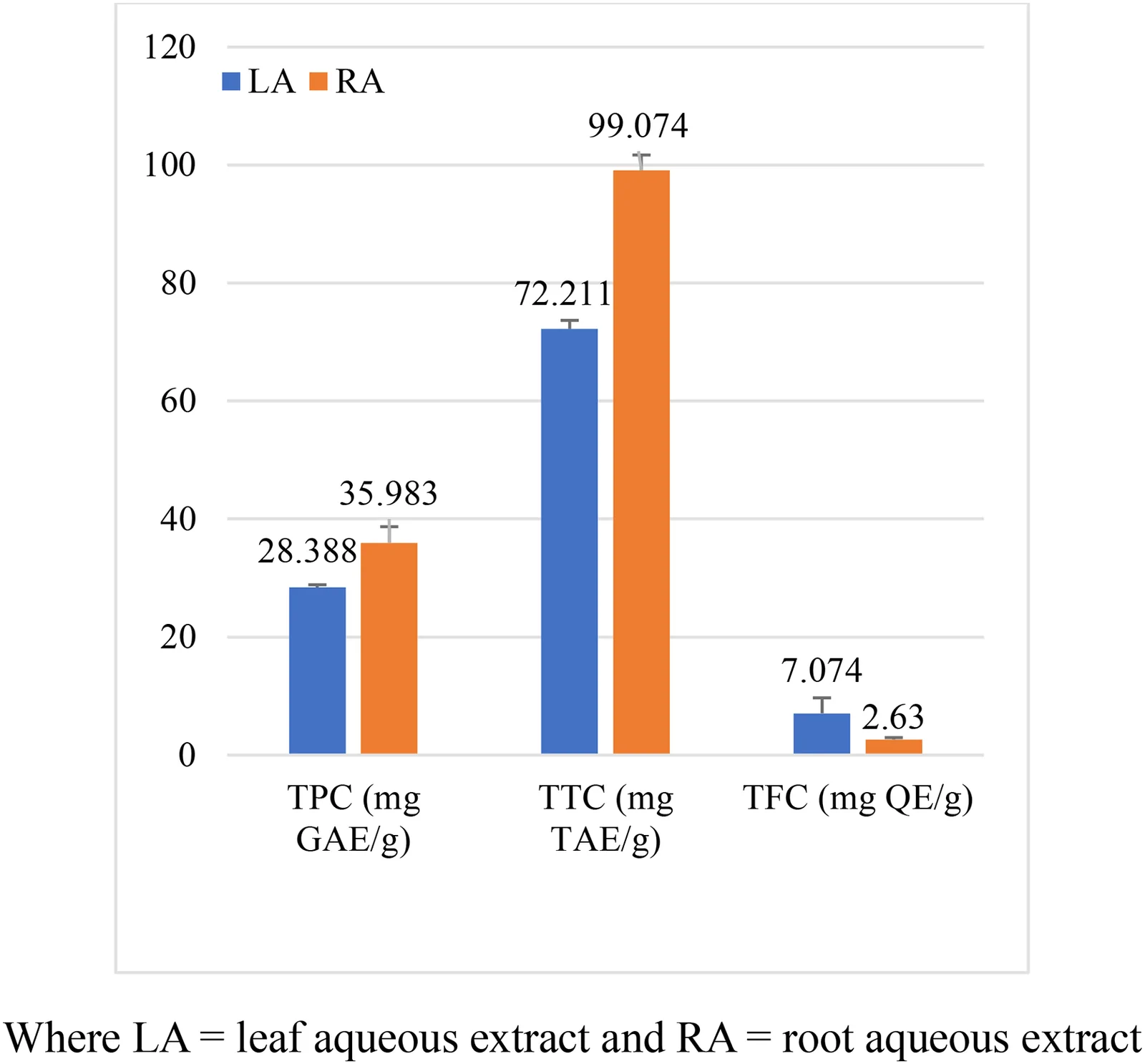
Comparative study of total phenolic content (TPC), total tannin content (TTC), and total flavonoid content (TFC) of two different aqueous extracts of *C. spinosa.*

Plants are primarily made up of polyphenols and flavonoids, which are potent antioxidants [31]. Based on Fig 14, RA contains a high level of phenolic and tannin compounds. The TPC and TTC values ranged from 28.39 ± 0.47 mg GAE/g and 72.21 ± 1.46 mg TAE/g in the LA to 35.98 ± 2.72 mg GAE/g and 99.07 ± 2.63 mg TAE/g in the RA. TFC was lower in the RA (2.63 ± 0.32 mg QE/g) compared to the LA (7.07 ± 2.63 mg QE/g).

### Antioxidant potential

**Fig 15:**
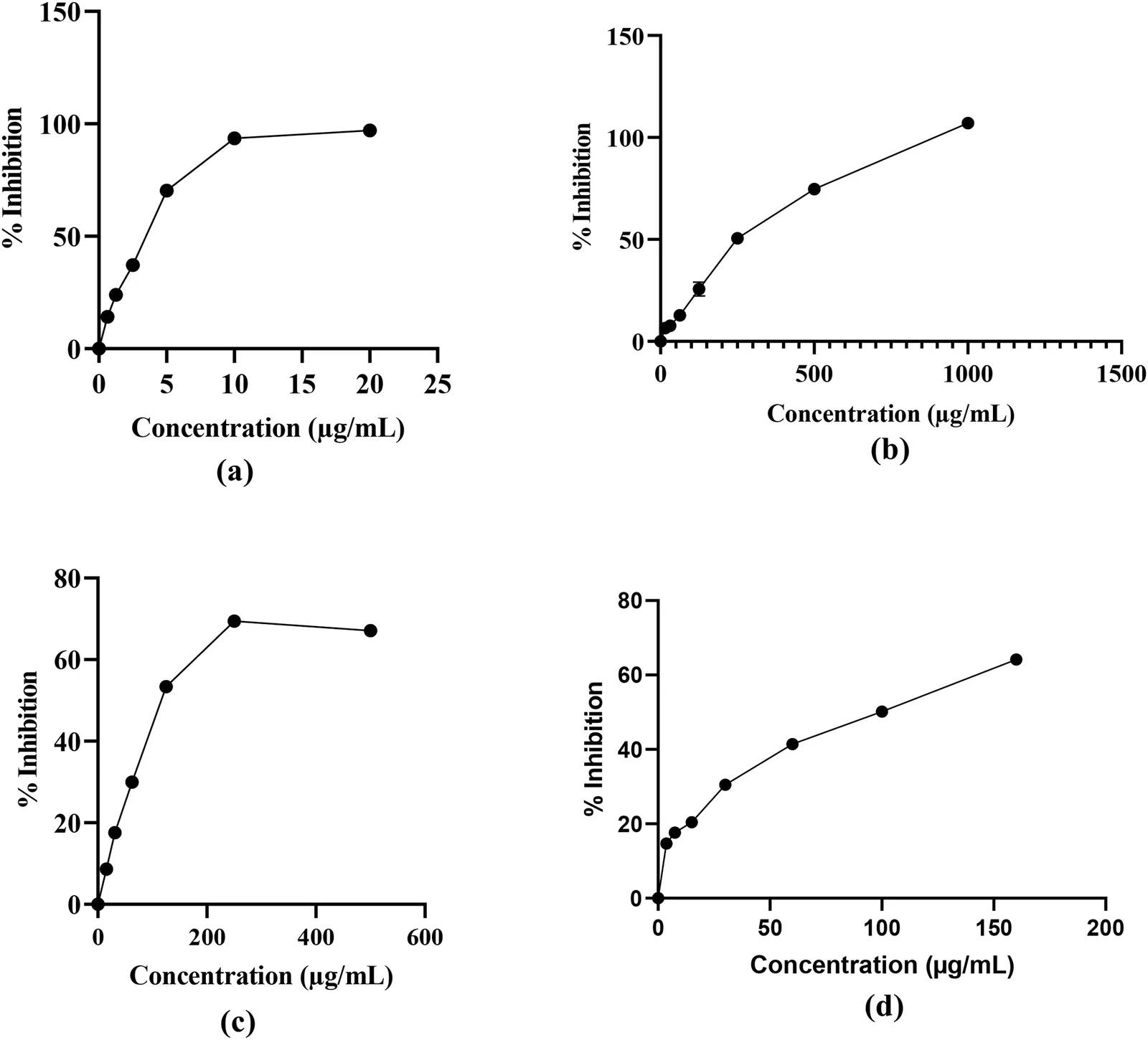
(a) Standard curve of DPPH inhibition by quercetin and plot of DPPH inhibition against concentration of (b) leaf aqueous extract (LA), (c) root aqueous extract (RA), and (d) leaf-assisted silver nanoparticles (L-AgNPs).

The antioxidant potential (IC_50_) of an antioxidant is the amount required to inhibit and evaluate its ability to scavenge DPPH by half [32]. Fig 15(a)–(d) presents a graphical representation of inhibition by standard Quercetin, aqueous extracts, and L-AgNPs. In this case, the IC_50_ of RA (65.66 ± 0.00 µg/mL) was lower than that of LA (723.20 ± 0.00 µg/mL), indicating that RA exhibited stronger antioxidant activity, likely due to its phenolic constituents. However, L-AgNPs showed a lower IC_50_ (106.10 ± 0.00 µg/mL) compared to R-AgNPs (>1000 µg/mL), suggesting higher antioxidant activity. Table 1 displays the IC_50_ values for standard Quercetin, aqueous extracts, and AgNPs.

**Table 1:** Antioxidant potential (IC_50_) of standard quercetin, aqueous extracts, and AgNPs.

S.N.	Nanoparticles/ Extracts	IC_50_ (µg/mL)
1.	Quercetin	3.83 ± 0.00
2.	LA	723.20 ± 0.00
3.	RA	65.66 ± 0.00
4.	L-AgNPs	106.10 ± 0.00
5.	R-AgNPs	>1000

Where, L-AgNPs = leaf-assisted synthesized silver nanoparticles, R-AgNPs = root-assisted synthesized silver nanoparticles, LA = leaf aqueous extract, and RA = root aqueous extract

### Antibacterial activity

**Fig 16:**
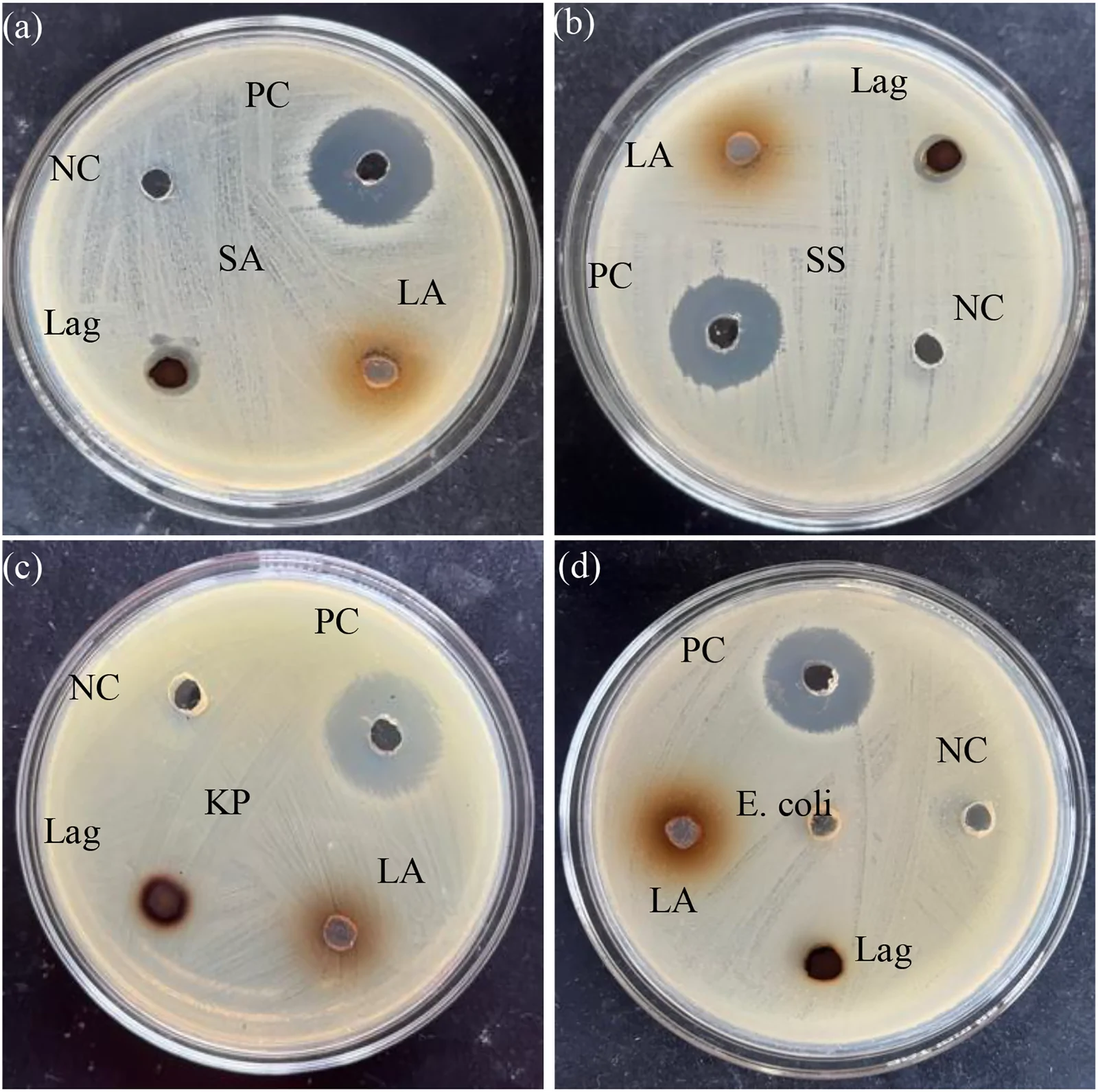
ZOI of leaf-assisted silver nanoparticles (L-AgNPs) against four pathogenic microorganisms. (a) *Staphylococcus aureus* (*SA*), (b) *Shigella sonnei* (*SS*), (c) *Klebsiella pneumoniae* (*KP*), and (d) *Escherichia coli* (*E. coli*).

**Fig 17:**
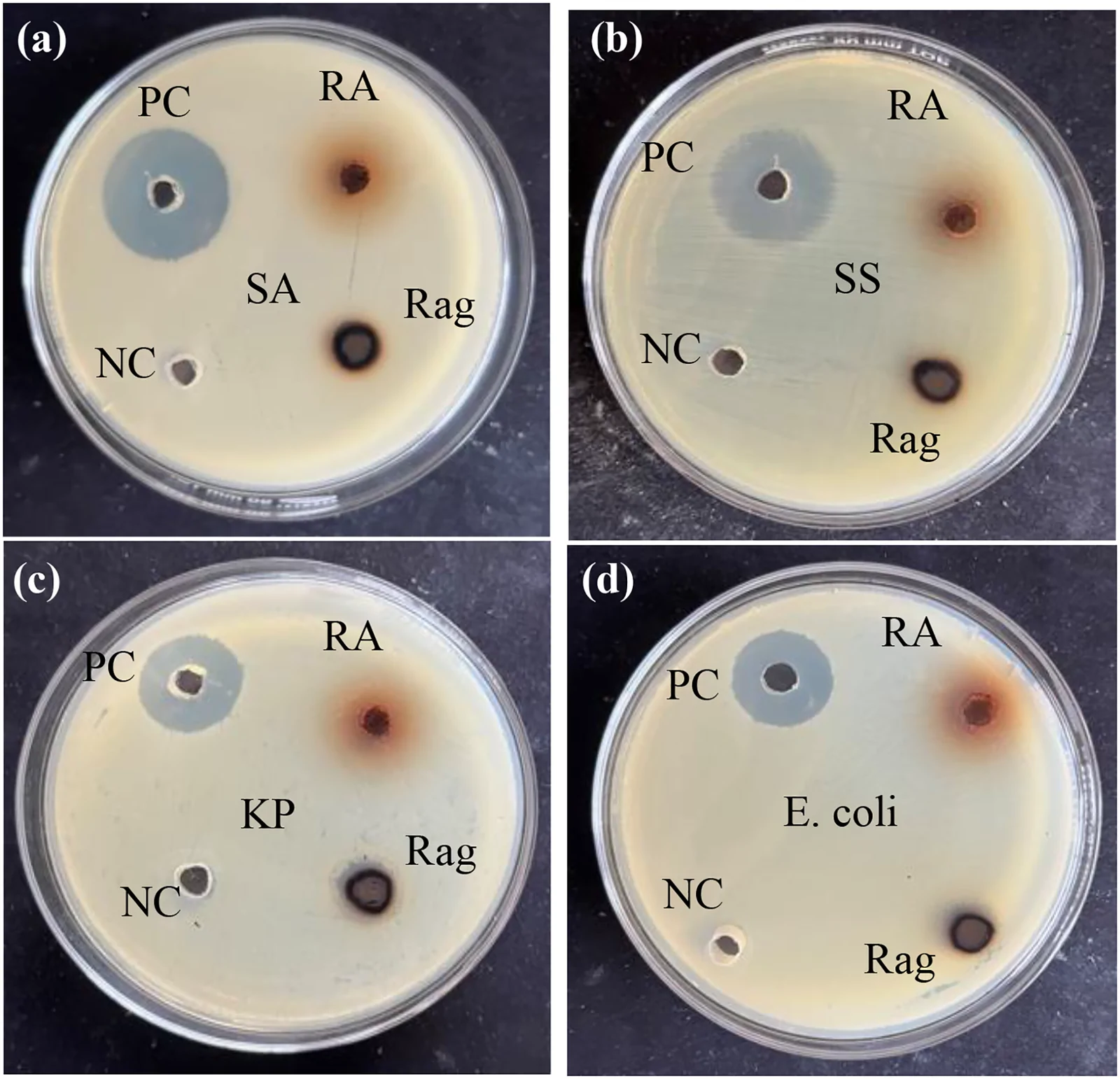
ZOI of root-assisted silver nanoparticles (R-AgNPs) against four pathogenic microorganisms. (a) *Staphylococcus aureus* (*SA*), (b) *Shigella sonnei* (*SS*), (c) *Klebsiella pneumoniae* (*KP*), and (d) *Escherichia coli* (*E. coli*).

Fig 16(a)–(d) and Fig 17(a)–(d) show a comparison of the zone of inhibition caused by AgNPs and their respective plant extract against the following human pathogens: *Staphylococcus aureus*, *Shigella sonnei*, *Klebsiella pneumoniae*, and *Escherichia coli*. The results indicated that both aqueous extracts displayed similar inhibition against all four bacteria (9–10 mm). R-AgNPs inhibited *Shigella sonnei* (12 mm), both *Staphylococcus aureus* and *Klebsiella pneumoniae* (13 mm), and *Escherichia coli* (11 mm). In comparison, L-AgNPs inhibited *Staphylococcus aureus* (14 mm), *Shigella sonnei* (13 mm), *Klebsiella pneumoniae* (13 mm), and *Escherichia coli* (10 mm). The antibacterial activity of the synthesized AgNPs along with respective crude extracts against these four pathogens is summarized in Table 2.

**Table 2:** Antibacterial screening of aqueous extracts and their respective AgNPs against four different pathogens.

Nanoparticles	Zone of inhibition (mm)			
	*Staphylococcus aureus*	*Shigella sonnei*	*Klebsiella pneumoniae*	*Escherichia coli*
LA	10	9	9	9
RA	10	9	9	9
L-AgNPs	14	13	13	10
R-AgNPs	13	12	13	11
Neomycin (Positive control)	21	22	22	21

Where, L-AgNPs = leaf-assisted synthesized silver nanoparticles, R-AgNPs = root-assisted synthesized silver nanoparticles, LA = leaf aqueous extract, and RA = root aqueous extract

### Minimum inhibitory concentration (MIC) and minimum bactericidal concentration (MBC)

**Fig 18:**
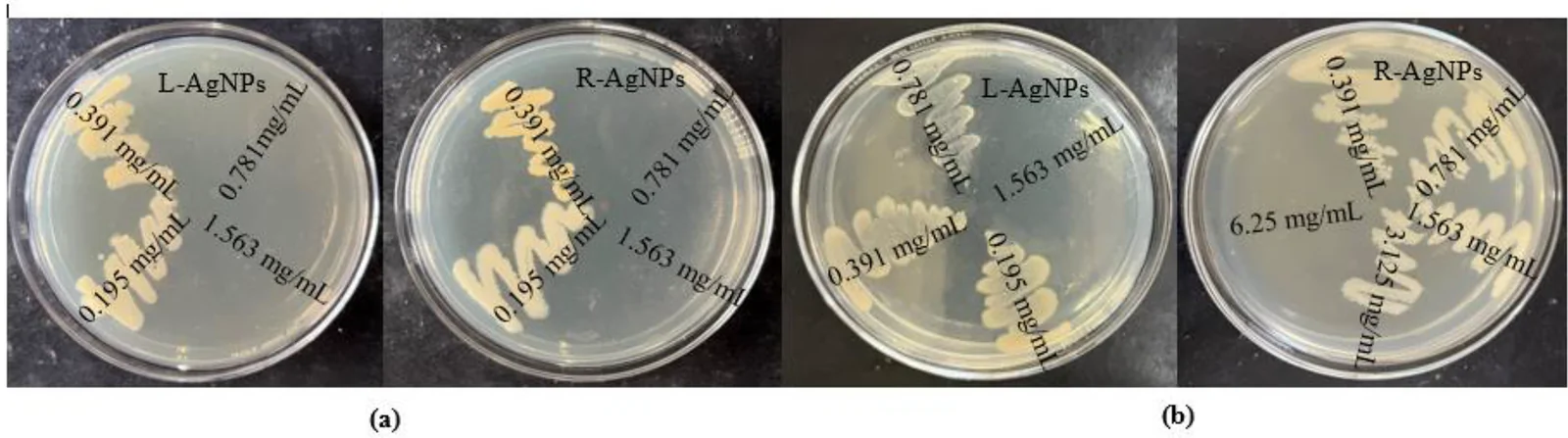
Nutrient agar plates showing the minimum bactericidal concentration of leaf-assisted silver nanoparticles (L-AgNPs) and root-assisted silver nanoparticles (R-AgNPs) against (a) *Staphylococcus aureus*, (b) *Klebsiella pneumoniae.*

Based on the data, L-AgNPs had a MIC of 0.391 mg/mL and an MBC of 0.781 mg/mL for both bacteria. R-AgNPs, however, had MICs of 3.125 and 0.391 mg/mL against *Klebsiella pneumoniae* and *Staphylococcus aureus*, respectively, with MBCs of 6.250 and 1.563 mg/mL. These results indicated that since the MIC and MBC of L-AgNPs are lower than those of R-AgNPs, L-AgNPs are more effective against the tested microorganisms. Table 3 displays the MIC and MBC for the synthesized AgNPs, and Fig 18(a) and (b) show nutrient agar plates with the MBC of L-AgNPs and R-AgNPs against *Staphylococcus aureus* and *Klebsiella pneumoniae*, respectively.

**Table 3:** MIC and MBC shown by L-AgNPs and R-AgNPs against *Staphylococcus aureus* and *Klebsiella pneumoniae.*

S.N.	Nanoparticles	MIC (mg/mL)		MBC (mg/mL)	
		*Staphylococcus aureus*	*Klebsiella pneumoniae*	*Staphylococcus aureus*	*Klebsiella* *pneumoniae*
1.	L-AgNPs	0.391	0.391	0.781	0.781
2.	R-AgNPs	0.391	3.125	1.563	6.250
3.	Neomycin (Positive control)	0.031	0.004	0.063	0.008

Where, L-AgNPs = leaf-assisted synthesized silver nanoparticles, R-AgNPs = root-assisted synthesized silver nanoparticles

### Toxicity

**Fig 19:**
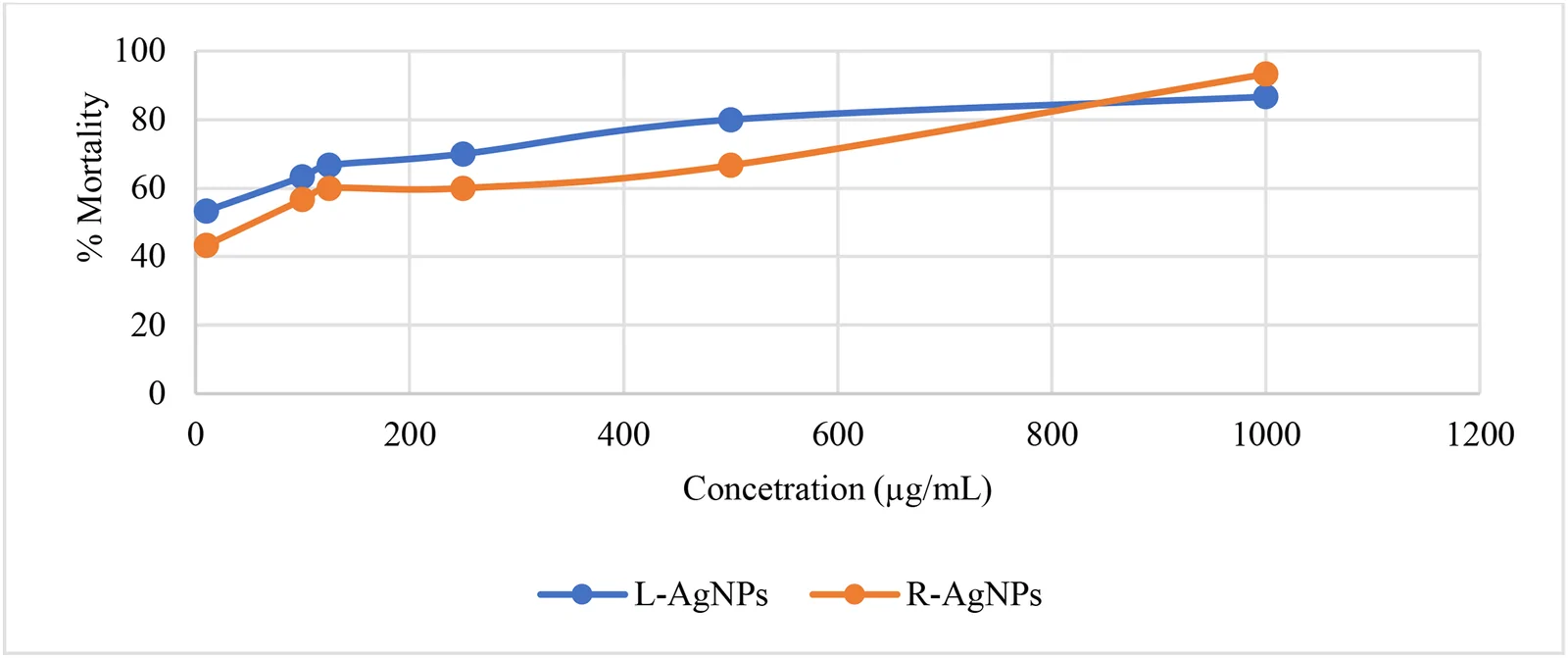
Plot of concentration vs % mortality of brine shrimp larvae for leaf-assisted silver nanoparticles (L-AgNPs) and root-assisted silver nanoparticles (R-AgNPs).

It is known that several plant secondary phytochemicals, including terpenes, alkaloids, and glycosides, can be toxic, and their NPs share this potential. Table 4 presents the toxicity of L-AgNPs and R-AgNPs in terms of LC_50_. Compared to the standard, L-AgNPs exhibit higher toxicity (LC_50_ = 12.31 ± 11.93 µg/mL) than R-AgNPs (LC_50_ = 40.24 ± 16.60 µg/mL). A documented decrease in the number of shrimp larvae remaining alive after 24 hours correlates with increased cytotoxicity as NPs concentration rises [33]. Fig 19 illustrates a graph of concentration versus percent mortality for synthesized AgNPs. The primary mechanism behind AgNPs’ toxicity has been described in the literature [34].

**Table 4:** Toxicity shown by the nanoparticles; L-AgNPs, and R-AgNPs with LC_50_.

S.N.	Nanoparticles	regression equation	LC_50_ (µg/mL)
1.	L-AgNPs	y = 0.4899x + 4.4659 R^2^ = 0.8872	12.31 ± 11.93
2.	R-AgNPs	y = 0.6618x + 3.9381 R^2^ = 0.6579	40.24 ± 16.60

Where, L-AgNPs = leaf-assisted synthesized silver nanoparticles, R-AgNPs = root-assisted synthesized silver nanoparticles

## Discussion

Modern nanotechnology relies heavily on the production of NPs, which are vital to technological advancement due to their versatility and enhanced activity compared to their parent materials. Their large surface area and nanoscale size give them unique physical and chemical properties [35]. This work aims to synthesize AgNPs with high antibacterial, antioxidant, and toxic effects from plant sources. Surface plasmon resonance caused AgNPs in aqueous solution to appear dark reddish-brown during incubation, suggesting that silver ions were reduced to silver particles upon exposure to plant extracts, as in [36].

UV-Vis spectroscopy provided further evidence of AgNP synthesis by showing a unique peak at 414 nm for L-AgNPs and 416 nm for R-AgNPs, consistent with earlier studies [37,38]. The FTIR spectrum showed C = C stretching at 1631 cm^-1^ and C-N and C-O bending at 1057–1064 cm^-1^, similar to previous research [39]. Additionally, there are Ag-O peaks at 1396–1399 cm^-1^ and Ag peaks at 874–881 cm^-1^, indicating the formation of AgNPs comparable to prior synthesis [38]. The XRD analysis revealed that the final sizes of L-AgNPs and R-AgNPs were 11.57 ± 0.35 nm and 10.05 ± 3.17 nm, respectively, aligning with the literature [38,40]. The diameters of L-AgNPs and R-AgNPs were measured at 38.79 ± 0.61 nm and 39.93 ± 0.83 nm, respectively, corresponding with previous findings [38].

This study showed that the decrease in weight of the fresh sample after drying is due to water loss from plant tissues. This finding indicates that plant materials have high moisture content and that the fresh-weight-dry-weight method is effective for determining moisture levels [41]. Also, the study identified glycosides, carbohydrates, alkaloids, polyphenols, and saponins in *C. spinosa*; other research found that the root lacked saponins and that the leaf extract lacked alkaloids [42]. Here, TTC ranged from 72.21 ± 1.46 mg TAE/g in the LA to 99.07 ± 2.63 mg TAE/g in the RA. TPC varied from 28.39 ± 0.47 mg GAE/g in the LA to 35.98 ± 2.72 mg GAE/g in the RA, indicating potential free radical scavenging activity. The TFC of the RA (2.63 ± 0.32 mg QE/g) was lower than that of the LA (7.07 ± 2.63 mg QE/g), although a previous study showed that the water-soluble leaf extract had higher TPC than the root extract [43]. Due to its higher TPC, RA may exhibit stronger antioxidant activity than LA, as indicated by the lower IC_50_. Compared to R-AgNPs, L-AgNPs had a lower IC_50_ of 106.10 ± 0.00 µg/mL. This suggests, consistent with previous research, that L-AgNPs possess higher antioxidant activity than R-AgNPs [37].

In the same way, AgNPs demonstrated much higher antibacterial activity than the extract alone, implying that plant biomolecules and AgNPs collaborate to enhance the NPs antimicrobial performance. Likewise, [39] studied that the AgNPs synthesized from roots proved effective against the Gram-positive bacterium *Staphylococcus aureus*, with a ZOI of 7.83 ± 0.17 mm (*Rhus chinensis* Mill), and the AgNPs generated from leaves were found to be effective against the Gram-negative bacterium *Klebsiella pneumoniae*, with a ZOI of 7.0 ± 0.29 mm. In this investigation, findings may be influenced by the size differences between L-AgNPs and R-AgNPs [44,45]. The MBC and MIC data showed that L-AgNPs were more effective against the tested bacteria, with lower MIC and MBC than R-AgNPs. Previous studies have looked at how AgNPs affect human pathogenic bacteria such as *Pseudomonas aeruginosa*, *Staphylococcus aureus*, and *Proteus mirabilis*, as well as *Escherichia coli*, *Klebsiella pneumoniae*, and *Bacillus subtilis*. The MICs ranged from 6.25 to 100 μg/mL, with zones of inhibition of 6–10 mm [46,47].

Finally, the toxicity of the NPs was assessed using the brine shrimp mortality assay. Compared to the standard, R-AgNPs are the least harmful (LC_50_ of 40.24 ± 16.60 µg/mL), while L-AgNPs are the most toxic (12.31 ± 11.93 µg/mL). Previous studies reported LC_50_ values of 13.50 µg/mL for *Brownlowia tersa* leaf-derived AgNPs [48] and 49.1 ± 2.33 µg/mL for *Elaeocarpus serratus* fruit -derived AgNPs [49]. These previous results indicate that AgNP toxicity varies with the plant part used, consistent with current research. In plant extracts, flavonoid content may predominantly influence antioxidant activity, while phenolic content plays a larger role in antibacterial effects in this study. Nevertheless, factors such as size, shape, chemical composition, and surface modifications of AgNPs, as well as their interactions with proteins, significantly affect their biological properties [50].

## Conclusion

Owing to the availability of phytochemicals, the study demonstrates that *C. spinosa* leaf and root extracts provide a rapid, easy, cost-effective, and environmentally friendly method for the formation of AgNPs. The DPPH assay demonstrated high antioxidant activity for L-AgNPs but low activity for R-AgNPs. Compared with extracts and R-AgNPs, L-AgNPs exhibited much superior antibacterial activity. The nano-silvers studied were efficient against bacteria at low concentrations and potent against both Gram-negative and Gram-positive bacteria. Furthermore, the toxicity assay in brine shrimp revealed dose-dependent effects from higher (1000 µg/mL) to lower (10 µg/mL) doses and also revealed that L-AgNPs were more lethal than R-AgNPs. Overall, the AgNPs investigated exhibit intriguing antioxidant, antibacterial, and toxic effects, suggesting that they may be viable antioxidant, antibacterial, and anticancer agents for use in the food and pharmaceutical industries in the future. However, further mechanistic and *in vivo* studies are required to elucidate their potential as therapeutic agents. Also, Future studies should focus on increasing synthesis volume and improving reaction conditions.

### Limitations of the study

Despite the promising results, this study has some limitations. No bioactive components were identified that specifically decreased or stabilized the NPs. Antibacterial efficacy was tested against a limited number of bacterial strains, but the underlying mechanisms of action were not investigated. Furthermore, *in vitro* studies were utilized to evaluate biological activity, but the brine shrimp mortality test offered only basic safety information. To determine the therapeutic potential of the NPs, further research should include precise phytochemical analysis, extensive biocompatibility assessments, mechanistic studies, and *in vivo* validation.

## Supporting information

S1 File (JPG)

S2 File (DOCX)
