# Supplementary material for: Phytochemical profiling-guided green synthesis and characterisation of silver nanoparticles using Catunaregam spinosa for biological applications
Source: PLoS One. 2026 Sep 2;21(9):e0356646. doi: 10.1371/journal.pone.0356646 (PMC13537541; doi:10.1371/journal.pone.0356646)
Supplement: Supplementary file 1 [file pone.0356646.s002.docx]

**Table S1: Absorbance of standard gallic acid, tannic acid, and Quercetin at different concentrations in triplicates**

| **Standards** | **Concentration (µg/mL)** | **Final absorbance** | | | **Initial absorbance** | | | **Calibration equation** |
| --- | --- | --- | --- | --- | --- | --- | --- | --- |
|  |  | A_1_ | A_2_ | A_3_ | A_1_ | A_2_ | A_3_ |  |
| Gallic acid | 100 | 1.516 | 1.424 | 1.499 | 0.422 | 0.394 | 0.408 | y = 0.0097x + 0.0043 R² = 0.98 |
|  | 80 | 1.025 | 0.985 | 0.975 | 0.309 | 0.298 | 0.304 |  |
|  | 60 | 0.783 | 0.774 | 0.814 | 0.253 | 0.233 | 0.252 |  |
|  | 30 | 0.455 | 0.464 | 0.451 | 0.155 | 0.158 | 0.155 |  |
|  | 15 | 0.262 | 0.271 | 0.276 | 0.106 | 0.106 | 0.106 |  |
|  | 7.5 | 0.165 | 0.171 | 0.168 | 0.074 | 0.079 | 0.078 |  |
|  | 3.75 | 0.112 | 0.114 | 0.113 | 0.058 | 0.06 | 0.059 |  |
| Tannic acid | 100 | 0.289 | 0.287 | 0.287 | 0.098 | 0.102 | 0.101 | y = 0.0018x + 0.012 R² = 0.9948 |
|  | 80 | 0.256 | 0.267 | 0.267 | 0.095 | 0.1 | 0.102 |  |
|  | 60 | 0.217 | 0.223 | 0.223 | 0.093 | 0.098 | 0.093 |  |
|  | 30 | 0.144 | 0.161 | 0.145 | 0.084 | 0.086 | 0.084 |  |
|  | 15 | 0.122 | 0.129 | 0.116 | 0.083 | 0.084 | 0.083 |  |
|  | 7.5 | 0.108 | 0.108 | 0.106 | 0.083 | 0.085 | 0.083 |  |
| Quercetin | 100 | 0.337 | 0.36 | 0.364 | 0.178 | 0.177 | 0.179 | y = 0.0018x + 0.0066 R² = 0.9944 |
|  | 80 | 0.333 | 0.33 | 0.331 | 0.179 | 0.176 | 0.175 |  |
|  | 60 | 0.264 | 0.263 | 0.26 | 0.158 | 0.15 | 0.153 |  |
|  | 30 | 0.19 | 0.196 | 0.191 | 0.127 | 0.129 | 0.125 |  |
|  | 15 | 0.142 | 0.14 | 0.144 | 0.109 | 0.098 | 0.106 |  |
|  | 7.5 | 0.109 | 0.112 | 0.111 | 0.09 | 0.101 | 0.093 |  |
|  | 3.75 | 0.12 | 0.121 | 0.122 | 0.103 | 0.103 | 0.104 |  |

**Absorbance of aqueous crude extracts in triplicate for estimation of total phenolic content, total tannin content, and total flavonoid content**

| **Extracts** | **Final absorbance** | | | **Initial absorbance** | | | **Phytochemical contents**  **(±SD)** | |
| --- | --- | --- | --- | --- | --- | --- | --- | --- |
|  | A_1_ | A_2_ | A_3_ | A_1_ | A_2_ | A_3_ |  |  |
| Leaf extract (LA) | 0.408 | 0.413 | 0.404 | 0.128 | 0.129 | 0.129 | 28.39 ± 0.47 | TPC  (mg GAE/g ) |
|  | 0.241 | 0.234 | 0.239 | 0.097 | 0.095 | 0.096 | 72.21 ± 1.46 | TTC  (mg TAE/g ) |
|  | 0.146 | 0.139 | 0.137 | 0.125 | 0.125 | 0.114 | 7.07 ± 2.63 | TFC  (mg QE/g) |
| Root extract (RA) | 0.492 | 0.454 | 0.511 | 0.131 | 0.13 | 0.136 | 35.98 ± 2.72 | TPC  (mg GAE/g ) |
|  | 0.294 | 0.291 | 0.283 | 0.102 | 0.097 | 0.098 | 99.07 ± 2.63 | TTC  (mg TAE/g ) |
|  | 0.153 | 0.154 | 0.155 | 0.142 | 0.143 | 0.143 | 2.63 ± 0.32 | TFC  (mg QE/g ) |

**Table S2: Antioxidant potential of aqueous crude extracts and nanoparticles (DPPH assay)**

| **Extracts/**  **Nanoparticles** | **Final absorbance** | | | **Initial absorbance** | | | **Concentration (µg/mL)** | **Control**  **(Water)** | **IC_50_ (µg/mL) (±SD)** |
| --- | --- | --- | --- | --- | --- | --- | --- | --- | --- |
|  | A_1_ | A_2_ | A_3_ | A_1_ | A_2_ | A_3_ |  |  |  |
| Leaf extract (LA) | 0.214 | 0.217 | 0.217 | 0.209 | 0.210 | 0.210 | 500 | 0.22867 | 723.20 ± 0.00 |
|  | 0.266 | 0.258 | 0.255 | 0.184 | 0.180 | 0.180 | 250 | 0.22867 |  |
|  | 0.326 | 0.322 | 0.305 | 0.166 | 0.166 | 0.164 | 125 | 0.22867 |  |
|  | 0.355 | 0.351 | 0.345 | 0.162 | 0.158 | 0.159 | 62.5 | 0.22867 |  |
|  | 0.369 | 0.367 | 0.355 | 0.159 | 0.157 | 0.157 | 31.25 | 0.22867 |  |
|  | 0.368 | 0.369 | 0.360 | 0.159 | 0.157 | 0.153 | 15.63 | 0.22867 |  |
| Root extract (RA) | 0.274 | 0.267 | 0.270 | 0.170 | 0.174 | 0.173 | 500 | 0.22867 | 65.66 ± 0.00 |
|  | 0.213 | 0.215 | 0.219 | 0.124 | 0.123 | 0.127 | 250 | 0.22867 |  |
|  | 0.240 | 0.247 | 0.245 | 0.106 | 0.106 | 0.103 | 125 | 0.22867 |  |
|  | 0.302 | 0.300 | 0.302 | 0.093 | 0.093 | 0.092 | 62.5 | 0.22867 |  |
|  | 0.332 | 0.331 | 0.334 | 0.087 | 0.086 | 0.087 | 31.25 | 0.22867 |  |
|  | 0.356 | 0.362 | 0.355 | 0.085 | 0.085 | 0.086 | 15.63 | 0.22867 |  |
| L-AgNPs | 0.275 | 0.274 | 0.273 | 0.159 | 0.161 | 0.160 | 100 | 0.22867 | 106.10 ± 0.00 |
|  | 0.256 | 0.257 | 0.255 | 0.174 | 0.174 | 0.172 | 80 | 0.22867 |  |
|  | 0.286 | 0.288 | 0.285 | 0.154 | 0.153 | 0.150 | 60 | 0.22867 |  |
|  | 0.275 | 0.275 | 0.273 | 0.114 | 0.117 | 0.115 | 30 | 0.22867 |  |
|  | 0.28 | 0.283 | 0.281 | 0.098 | 0.099 | 0.101 | 15 | 0.22867 |  |
|  | 0.276 | 0.279 | 0.278 | 0.090 | 0.088 | 0.090 | 7.5 | 0.22867 |  |
|  | 0.284 | 0.280 | 0.281 | 0.086 | 0.086 | 0.088 | 3.75 | 0.22867 |  |
| R-AgNPs | 0.274 | 0.269 | 0.267 | 0.127 | 0.126 | 0.127 | 100 | 0.22867 | >1000 |
|  | 0.302 | 0.282 | 0.271 | 0.112 | 0.113 | 0.113 | 80 | 0.22867 |  |
|  | 0.288 | 0.291 | 0.287 | 0.106 | 0.107 | 0.107 | 60 | 0.22867 |  |
|  | 0.290 | 0.294 | 0.293 | 0.093 | 0.094 | 0.096 | 30 | 0.22867 |  |
|  | 0.284 | 0.282 | 0.283 | 0.087 | 0.086 | 0.087 | 15 | 0.22867 |  |
|  | 0.293 | 0.295 | 0.294 | 0.087 | 0.086 | 0.085 | 7.50 | 0.22867 |  |
|  | 0.295 | 0.296 | 0.298 | 0.085 | 0.085 | 0.084 | 3.75 | 0.22867 |  |

**Table S3:Toxicity analysis (Brine shrimp lethality assay)**

| **Nanoparticles** | **Concentration**  **(µg/mL)** | **No. of dead nauplii** | | | **Total dead nauplii** | **Total nauplii** | **% Mortality** | **LC_50_ (µg/mL)**  **(±SD)** |
| --- | --- | --- | --- | --- | --- | --- | --- | --- |
| L-AgNPs | 1000 | 9 | 8 | 9 | 26 | 30 | 86.67 | 12.31 ± 11.93 |
|  | 500 | 8 | 9 | 7 | 24 | 30 | 80 |  |
|  | 250 | 7 | 8 | 6 | 21 | 30 | 70 |  |
|  | 125 | 5 | 7 | 8 | 20 | 30 | 66.67 |  |
|  | 100 | 6 | 6 | 7 | 19 | 30 | 63.33 |  |
|  | 10 | 6 | 6 | 4 | 16 | 30 | 53.33 |  |
| R-AgNPs | 1000 | 10 | 10 | 8 | 28 | 30 | 93.33 | 40.24 ± 16.60 |
|  | 500 | 7 | 8 | 5 | 20 | 30 | 66.67 |  |
|  | 250 | 6 | 6 | 6 | 18 | 30 | 60 |  |
|  | 125 | 5 | 6 | 7 | 18 | 30 | 60 |  |
|  | 100 | 6 | 6 | 5 | 17 | 30 | 56.67 |  |
|  | 10 | 4 | 5 | 4 | 13 | 30 | 43.33 |  |
