# Supplementary material for: Phytochemical profiling-guided green synthesis and characterisation of silver nanoparticles using Catunaregam spinosa for biological applications
Source: PLoS One. 2026 Sep 2;21(9):e0356646. doi: 10.1371/journal.pone.0356646 (PMC13537541; doi:10.1371/journal.pone.0356646)
Supplement: Supplementary file 2 [file pone.0356646.s005.docx]

Please include the following items when submitting your revised manuscript:

- A letter that responds to each point raised by the academic editor and reviewer(s). You should upload this letter as a separate file labeled 'Response to Reviewers'.
- A marked-up copy of your manuscript that highlights changes made to the original version. You should upload this as a separate file labeled 'Revised Manuscript with Track Changes'.
- An unmarked version of your revised paper without tracked changes. You should upload this as a separate file labeled 'Manuscript'.

If you would like to make changes to your financial disclosure, please include your updated statement in your cover letter. Guidelines for resubmitting your figure files are available below the reviewer comments at the end of this letter.

Thank you very much for the constructive comments and suggestions on the manuscript. I have addressed the comments raised by the reviewers and incorporated in the revised manuscript as follows:

Reviewer #1: 1. This suggestion is not carried out properly and it should be rectified (Check the abbreviations throughout the manuscript and introduce the abbreviation when the full word appears The first time in the abstract and the remaining for the text and then use only the abbreviation (For example, NPs, silver nanoparticles (AgNPs), and these types of corrections need to be checked all other abbreviations used in the manuscript.

Ans: The manuscript has been thoroughly examined to verify that any abbreviations are used only after the full term appears for the first time in both the Abstract and the main body. After that, only abbreviations were used regularly throughout the work. Appropriate corrections, including the presentation of words like silver nanoparticles (AgNPs) and other abbreviations, have been made where applicable.

2. The authors have given the statement without reference and the same should be added and the sentence may be shifted to another part of materials and methods from statistical investigation (The authors may justify how the dose optimized for the present investigation in the materials and methods (either based on available literature or previous study). If from the others study should be supported with reference).

Ans: The Materials and Methods section has been edited to state that the experimental approach, including the selected dose, was based on previously published standard methodologies. Appropriate references for the experimental technique and dose selection have been included. In addition, the relevant information has been transferred from the Statistical Analysis section to the appropriate subsection in the Materials and Methods section to improve the manuscript's organization and clarity.

3. The the authors may also include the limitation of the present findings for a better understanding of the manuscript.

Ans: A paragraph addressing the limits of the current study has been added to provide a fair assessment of the findings and assist in a better understanding of the breadth and relevance of the results.
