# Supplementary material for: Phytochemical profiling-guided green synthesis and characterisation of silver nanoparticles using Catunaregam spinosa for biological applications
Source: PLoS One. 2026 Sep 2;21(9):e0356646. doi: 10.1371/journal.pone.0356646 (PMC13537541; doi:10.1371/journal.pone.0356646)
Supplement: Peer review history

# Peer review: Decision Letter 0

**DOI:** 10.1371/journal.pone.0356646.r001

**Authors:** Salem Salem

15 Feb 2026

Phytochemical Analysis and Synthesis of Silver Nanoparticles Using Catunaregam spinosa (Thunb.) Triveng for Antioxidant and Antibacterial Activities

PLOS One

Dear Dr. Sharma,

Thank you for submitting your manuscript to PLOS ONE. After careful consideration, we feel that it has merit but does not fully meet PLOS ONE’s publication criteria as it currently stands. Therefore, we invite you to submit a revised version of the manuscript that addresses the points raised during the review process.

==============================

**ACADEMIC EDITOR:  Major Revision**

Please submit your revised manuscript by Apr 01 2026 11:59PM. If you will need more time than this to complete your revisions, please reply to this message or contact the journal office at plosone@plos.org. When you're ready to submit your revision, log on to https://www.editorialmanager.com/pone/ and select the 'Submissions Needing Revision' folder to locate your manuscript file.

- A letter that responds to each point raised by the academic editor and reviewer(s). You should upload this letter as a separate file labeled 'Response to Reviewers'.
- A marked-up copy of your manuscript that highlights changes made to the original version. You should upload this as a separate file labeled 'Revised Manuscript with Track Changes'.
- An unmarked version of your revised paper without tracked changes. You should upload this as a separate file labeled 'Manuscript'.

If you would like to make changes to your financial disclosure, please include your updated statement in your cover letter. Guidelines for resubmitting your figure files are available below the reviewer comments at the end of this letter.

If applicable, we recommend that you deposit your laboratory protocols in protocols.io to enhance the reproducibility of your results. Protocols.io assigns your protocol its own identifier (DOI) so that it can be cited independently in the future. For instructions see: https://journals.plos.org/plosone/s/submission-guidelines#loc-laboratory-protocols. Additionally, PLOS ONE offers an option for publishing peer-reviewed Lab Protocol articles, which describe protocols hosted on protocols.io. Read more information on sharing protocols at https://plos.org/protocols?utm_medium=editorial-email&utm_source=authorletters&utm_campaign=protocols.

We look forward to receiving your revised manuscript.

Kind regards,

Salem S. Salem

Academic Editor

PLOS One

Journal Requirements:

When submitting your revision, we need you to address these additional requirements.

1. Please ensure that your manuscript meets PLOS ONE's style requirements, including those for file naming. The PLOS ONE style templates can be found at

https://journals.plos.org/plosone/s/file?id=wjVg/PLOSOne_formatting_sample_main_body.pdf and

https://journals.plos.org/plosone/s/file?id=ba62/PLOSOne_formatting_sample_title_authors_affiliations.pdf

2. In your Methods section, please provide additional information regarding the permits you obtained for the work. Please ensure you have included the full name of the authority that approved the field site access and, if no permits were required, a brief statement explaining why

3. Thank you for stating the following in your Competing Interests section:

“No competing interest”

Please complete your Competing Interests on the online submission form to state any Competing Interests. If you have no competing interests, please state "The authors have declared that no competing interests exist.", as detailed online in our guide for authors at http://journals.plos.org/plosone/s/submit-now

This information should be included in your cover letter; we will change the online submission form on your behalf.

4. Please note that your Data Availability Statement is currently missing [the repository name and/or the DOI/accession number of each dataset OR a direct link to access each database]. If your manuscript is accepted for publication, you will be asked to provide these details on a very short timeline. We therefore suggest that you provide this information now, though we will not hold up the peer review process if you are unable.

5. If the reviewer comments include a recommendation to cite specific previously published works, please review and evaluate these publications to determine whether they are relevant and should be cited. There is no requirement to cite these works unless the editor has indicated otherwise.

[Note: HTML markup is below. Please do not edit.]

Reviewers' comments:

Reviewer's Responses to Questions

**Comments to the Author**

1. Is the manuscript technically sound, and do the data support the conclusions?

Reviewer #1: Partly

Reviewer #2: Partly

**********

2. Has the statistical analysis been performed appropriately and rigorously?

Reviewer #1: Yes

Reviewer #2: No

**********

3. Have the authors made all data underlying the findings in their manuscript fully available?

The PLOS Data policy

Reviewer #1: Yes

Reviewer #2: No

**********

4. Is the manuscript presented in an intelligible fashion and written in standard English?

Reviewer #1: Yes

Reviewer #2: No

**********

Reviewer #1: 1. There are some grammatical, alignments and typographical errors are noted in the manuscript and it should be thoroughly checked and corrected throughout the manuscript.

2. The use of abbreviations in the abstract may distract readers who wish to quickly skim through several publications before deciding to read one in full. It may therefore help to write out terms fully in abstract (For example, FTIR, XRD, etc.,).

3. Check the abbreviations throughout the manuscript and introduce the abbreviation when the full word appears The first time in the abstract and the remaining for the text and then use only the abbreviation (For example, nanoparticle (NP), AgNPs, minimum bactericidal concentration (MBC), etc.,). Make a word abbreviated in the article that is repeated at least three times in the text, not all words to be abbreviated.

4. The full form of the species should be given when the first time appears in both the abstract and in the remaining part of the manuscript and it should be followed by only the first letter of the genus (For example, Catunaregam spinosa when the first time appear and followed by C. spinosa) and this should be checked all other species used in the manuscript.

5. The authors may include the details of the quantity of fresh sample used and the quantity of the powder obtained (the ratio between fresh plant and powder) under the heading materials and methods.

6. The authors should mention the following when referring to GraphPad Prism version 8.0.2 (GraphPad Software, San Diego, CA, USA).

7. The authors should provide more information in the figure legends for non-experts. All legends should have enough description for a reader to understand the figure without having to refer back to the main text of the manuscript. For example, the necessary expansion (for abbreviations), should be given which are used in the present investigation.

8. The authors may justify how the dose optimized for the present investigation in the materials and methods (either based on available literature or previous study). If from the others study should be supported with reference.

9. The technical terms (Latin Phrase) "in vivo" should be italic and it should be checked all over the manuscript.

10. The conclusion provides a comprehensive overview of the review, but it could be more concise. Additionally, the conclusion could be strengthened by explicitly stating the limitation and implications of the data/review.

Reviewer #2: The manuscript presents the green synthesis of silver nanoparticles by Catunaregam spinosa leaf and root extracts, as well as the antioxidant, antibacterial, and cytotoxic activities of the synthesized silver nanoparticles. The topic of the manuscript is appropriate for nanobiotechnology and green synthesis of nanoparticles. The manuscript presents several characterization methods, which is good. However, the manuscript needs major changes before it can be considered technically sound and suitable for publication.

The manuscript deals with a relevant topic, which has potential. However, the paper requires major revision. Improvement in the statistical analysis, data interpretation, and clarity of writing is needed before the paper can be considered for publication.

**********

what does this mean?). If published, this will include your full peer review and any attached files.

If you choose “no”, your identity will remain anonymous but your review may still be made public.

**Do you want your identity to be public for this peer review?**  For information about this choice, including consent withdrawal, please see our Privacy Policy

Reviewer #1: No

Reviewer #2: **Yes:** Rezhna kheder ali

**********

[NOTE: If reviewer comments were submitted as an attachment file, they will be attached to this email and accessible via the submission site. Please log into your account, locate the manuscript record, and check for the action link "View Attachments". If this link does not appear, there are no attachment files.]

To ensure your figures meet our technical requirements, please review our figure guidelines: https://journals.plos.org/plosone/s/figures

You may also use PLOS’s free figure tool, NAAS, to help you prepare publication quality figures: https://journals.plos.org/plosone/s/figures#loc-tools-for-figure-preparation.

NAAS will assess whether your figures meet our technical requirements by comparing each figure against our figure specifications.

# Author comment: Author response to Decision Letter 1

**DOI:** 10.1371/journal.pone.0356646.r002

23 Mar 2026

All the comments/suggestions raised by the reviewers has been incorporated in the revised manuscript.

When submitting your revision, we need you to address these additional requirements.

1. Please ensure that your manuscript meets PLOS ONE's style requirements, including those for file naming. The PLOS ONE style templates can be found at

https://journals.plos.org/plosone/s/file?id=wjVg/PLOSOne_formatting_sample_main_body.pdf and

https://journals.plos.org/plosone/s/file?id=ba62/PLOSOne_formatting_sample_title_authors_affiliations.pdf

2. In your Methods section, please provide additional information regarding the permits you obtained for the work. Please ensure you have included the full name of the authority that approved the field site access and, if no permits were required, a brief statement explaining why

3. Thank you for stating the following in your Competing Interests section:

“No competing interest”

Please complete your Competing Interests on the online submission form to state any Competing Interests. If you have no competing interests, please state "The authors have declared that no competing interests exist.", as detailed online in our guide for authors at http://journals.plos.org/plosone/s/submit-now

This information should be included in your cover letter; we will change the online submission form on your behalf.

4. Please note that your Data Availability Statement is currently missing [the repository name and/or the DOI/accession number of each dataset OR a direct link to access each database]. If your manuscript is accepted for publication, you will be asked to provide these details on a very short timeline. We therefore suggest that you provide this information now, though we will not hold up the peer review process if you are unable.

5. If the reviewer comments include a recommendation to cite specific previously published works, please review and evaluate these publications to determine whether they are relevant and should be cited. There is no requirement to cite these works unless the editor has indicated otherwise.

[Note: HTML markup is below. Please do not edit.]

Reviewers' comments:

Reviewer's Responses to Questions

Comments to the Author

1. Is the manuscript technically sound, and do the data support the conclusions?

The manuscript must describe a technically sound piece of scientific research with data that supports the conclusions. Experiments must have been conducted rigorously, with appropriate controls, replication, and sample sizes. The conclusions must be drawn appropriately based on the data presented.

Reviewer #1: Partly

Reviewer #2: Partly

________________________________________

2. Has the statistical analysis been performed appropriately and rigorously?

Reviewer #1: Yes

Reviewer #2: No

________________________________________

3. Have the authors made all data underlying the findings in their manuscript fully available?

The PLOS Data policy requires authors to make all data underlying the findings described in their manuscript fully available without restriction, with rare exception (please refer to the Data Availability Statement in the manuscript PDF file). The data should be provided as part of the manuscript or its supporting information, or deposited to a public repository. For example, in addition to summary statistics, the data points behind means, medians and variance measures should be available. If there are restrictions on publicly sharing data—e.g. participant privacy or use of data from a third party—those must be specified.

Reviewer #1: Yes

Reviewer #2: No

________________________________________

4. Is the manuscript presented in an intelligible fashion and written in standard English?

PLOS ONE does not copyedit accepted manuscripts, so the language in submitted articles must be clear, correct, and unambiguous. Any typographical or grammatical errors should be corrected at revision, so please note any specific errors here.

Reviewer #1: Yes

Reviewer #2: No

________________________________________

5. Review Comments to the Author

Please use the space provided to explain your answers to the questions above. You may also include additional comments for the author, including concerns about dual publication, research ethics, or publication ethics. (Please upload your review as an attachment if it exceeds 20,000 characters)

Reviewer #1: 1. There are some grammatical, alignment, and typographical errors noted in the manuscript, and it should be thoroughly checked and corrected throughout the manuscript.

Ans: Grammatical, alignments, and typographical errors have been thoroughly checked and corrected throughout the revised manuscript.

2. The use of abbreviations in the abstract may distract readers who wish to quickly skim through several publications before deciding to read one in full. It may therefore help to write out terms fully in abstract (For example, FTIR, XRD, etc.).

Ans: Full terms instead of abbreviations have been used in the abstract. For example, FTIR, XRD, FE-SEM, EDX, etc.

3. Check the abbreviations throughout the manuscript and introduce the abbreviation when the full word appears, the first time in the abstract and the remaining for the text, and then use only the abbreviation (For example, nanoparticle (NP), AgNPs, minimum bactericidal concentration (MBC), etc.). Make a word abbreviated in the article that is repeated at least three times in the text, not all words to be abbreviated.

Ans: The abbreviations have been checked throughout the manuscript, and introduced wherever the full word appears except the first time in the abstract and the remaining for the text. (For example, aqueous leaf extract (LA), aqueous root extract (RA), nanoparticles (NPs), silver nanoparticles (AgNPs), leaf-assisted silver nanoparticles (L-AgNPs), root-assisted silver nanoparticles (R-AgNPs), minimum inhibitory concentration (MIC), minimum bactericidal concentration (MBC), etc.

4. The full form of the species should be given when the first time appears in both the abstract and in the remaining part of the manuscript and it should be followed by only the first letter of the genus (For example, Catunaregam spinosa when the first time appear and followed by C. spinosa) and this should be checked all other species used in the manuscript.

Ans: The full form and abbreviations have been thoroughly reviewed for all species used in the manuscript, as stated.

5. The authors may include the details of the quantity of fresh sample used and the quantity of the powder obtained (the ratio between fresh plant and powder) under the heading materials and methods.

Ans: The details of the quantity of fresh sample used and the quantity of the powder obtained have been added in the materials and methods section, giving the new title ′Determination of moisture content′, and the finding indicates that plant materials have high moisture content and that the fresh-weight-dry-weight method is effective for determining moisture levels.

6. The authors should mention the following when referring to GraphPad Prism version 8.0.2 (GraphPad Software, San Diego, CA, USA).

Ans: As suggested, the name of the publisher/organizer of the software used, and the location have been added to the manuscript. E.g., GraphPad Prism 8.0.2 (GraphPad Software Inc., Boston, Massachusetts, USA), OriginPro 2019b (OriginLab Corporation, Northampton, Massachusetts, USA), ImageJ 1.54g (National Institutes of Health, Bethesda, Maryland, USA), etc.

7. The authors should provide more information in the figure legends for non-experts. All legends should have enough description for a reader to understand the figure without having to refer back to the main text of the manuscript. For example, the necessary expansion (for abbreviations) should be given which are used in the present investigation.

Ans: Figure legends have been rechecked and improved for ease of non-experts, including enough description for a reader to understand the figure without referring back to the main text of the manuscript.

8. The authors may justify how the dose optimized for the present investigation in the materials and methods (either based on available literature or a previous study). If from the others study should be supported with reference.

Ans: The dose optimized for the present investigation in the materials and methods was based on available literature, which has been presented in the reference section.

9. The technical terms (Latin Phrase) "in vivo" should be italic and it should be checked all over the manuscript.

Ans: The technical term (Latin Phrase) "in vivo" has been italicized all over the manuscript.

10. The conclusion provides a comprehensive overview of the review, but it could be more concise. Additionally, the conclusion could be strengthened by explicitly stating the limitation and implications of the data/review.

Ans: The conclusion has been revised to improve conciseness and to explicitly address the limitations and implications of the data/review as suggested.

Reviewer #2: The manuscript presents the green synthesis of silver nanoparticles by Catunaregam spinosa leaf and root extracts, as well as the antioxidant, antibacterial, and cytotoxic activities of the synthesized silver nanoparticles. The topic of the manuscript is appropriate for nanobiotechnology and green synthesis of nanoparticles. The manuscript presents several characterization methods, which is good. However, the manuscript needs major changes before it can be considered technically sound and suitable for publication.

The manuscript deals with a relevant topic, which has potential. However, the paper requires major revision. Improvement in the statistical analysis, data interpretation, and clarity of writing is needed before the paper can be considered for publication.

Ans: The manuscript has been enhanced in the statistical analysis; data interpretation has been reviewed, improvised and writing has been done with more clarity and fluency accordingly.

# Peer review: Decision Letter 1

**DOI:** 10.1371/journal.pone.0356646.r003

**Authors:** Salem Salem

24 Jun 2026

Dear Dr. Sharma,

Thank you for submitting your manuscript to PLOS ONE. After careful consideration, we feel that it has merit but does not fully meet PLOS ONE’s publication criteria as it currently stands. Therefore, we invite you to submit a revised version of the manuscript that addresses the points raised during the review process.

==============================

**ACADEMIC EDITOR:**

Please submit your revised manuscript by Aug 08 2026 11:59PM. If you will need more time than this to complete your revisions, please reply to this message or contact the journal office at plosone@plos.org. When you're ready to submit your revision, log on to https://www.editorialmanager.com/pone/ and select the 'Submissions Needing Revision' folder to locate your manuscript file.

- A letter that responds to each point raised by the academic editor and reviewer(s). You should upload this letter as a separate file labeled 'Response to Reviewers'.
- A marked-up copy of your manuscript that highlights changes made to the original version. You should upload this as a separate file labeled 'Revised Manuscript with Track Changes'.
- An unmarked version of your revised paper without tracked changes. You should upload this as a separate file labeled 'Manuscript'.

If you would like to make changes to your financial disclosure, please include your updated statement in your cover letter. Guidelines for resubmitting your figure files are available below the reviewer comments at the end of this letter.

If applicable, we recommend that you deposit your laboratory protocols in protocols.io to enhance the reproducibility of your results. Protocols.io assigns your protocol its own identifier (DOI) so that it can be cited independently in the future. For instructions see: https://journals.plos.org/plosone/s/submission-guidelines#loc-laboratory-protocols. Additionally, PLOS ONE offers an option for publishing peer-reviewed Lab Protocol articles, which describe protocols hosted on protocols.io. Read more information on sharing protocols at https://plos.org/protocols?utm_medium=editorial-email&utm_source=authorletters&utm_campaign=protocols.

As the corresponding author, your ORCID iD is verified in the submission system and will appear in the published article. PLOS supports the use of ORCID, and we encourage all coauthors to register for an ORCID iD and use it as well. Please encourage your coauthors to verify their ORCID iD within the submission system before final acceptance, as unverified ORCID iDs will not appear in the published article. *Only*  the individual author can complete the verification step; PLOS staff *cannot*  verify ORCID iDs on behalf of authors.

We look forward to receiving your revised manuscript.

Kind regards,

Salem S. Salem

Academic Editor

PLOS One

Journal Requirements:

If the reviewer comments include a recommendation to cite specific previously published works, please review and evaluate these publications to determine whether they are relevant and should be cited. There is no requirement to cite these works unless the editor has indicated otherwise.

Please review your reference list to ensure that it is complete and correct. If you have cited papers that have been retracted, please include the rationale for doing so in the manuscript text, or remove these references and replace them with relevant current references. Any changes to the reference list should be mentioned in the rebuttal letter that accompanies your revised manuscript. If you need to cite a retracted article, indicate the article’s retracted status in the References list and also include a citation and full reference for the retraction notice.

[Note: HTML markup is below. Please do not edit.]

Reviewers' comments:

Reviewer's Responses to Questions

**Comments to the Author**

Reviewer #1: All comments have been addressed

**********

2. Is the manuscript technically sound, and do the data support the conclusions?

Reviewer #1: Partly

**********

3. Has the statistical analysis been performed appropriately and rigorously?

Reviewer #1: Yes

**********

4. Have the authors made all data underlying the findings in their manuscript fully available?

The PLOS Data policy

Reviewer #1: Yes

**********

5. Is the manuscript presented in an intelligible fashion and written in standard English?

Reviewer #1: Yes

**********

Reviewer #1: 1. This suggestion is not carried out properly and it should be rectified (Check the abbreviations throughout the manuscript and introduce the abbreviation when the full word appears The first time in the abstract and the remaining for the text and then use only the abbreviation (For example, NPs, silver nanoparticles (AgNPs), and these types of corrections need to be checked all other abbreviations used in the manuscript.

2. The authors have given the statement without reference and the same should be added and the sentence may be shifted to another part of materials and methods from statistical investigation (The authors may justify how the dose optimized for the present investigation in the materials and methods (either based on available literature or previous study). If from the others study should be supported with reference).

3. The the authors may also include the limitation of the present findings for a better understanding of the manuscript.

**********

what does this mean?). If published, this will include your full peer review and any attached files.

If you choose “no”, your identity will remain anonymous but your review may still be made public.

**Do you want your identity to be public for this peer review?**  For information about this choice, including consent withdrawal, please see our Privacy Policy

Reviewer #1: No

**********

[NOTE: If reviewer comments were submitted as an attachment file, they will be attached to this email and accessible via the submission site. Please log into your account, locate the manuscript record, and check for the action link "View Attachments". If this link does not appear, there are no attachment files.]

To ensure your figures meet our technical requirements, please review our figure guidelines: https://journals.plos.org/plosone/s/figures

You may also use PLOS’s free figure tool, NAAS, to help you prepare publication quality figures: https://journals.plos.org/plosone/s/figures#loc-tools-for-figure-preparation.

NAAS will assess whether your figures meet our technical requirements by comparing each figure against our figure specifications.

# Author comment: Author response to Decision Letter 2

**DOI:** 10.1371/journal.pone.0356646.r004

11 Jul 2026

The comments and suggestion raised by the editor and reviewers has been incorporated in the revised manuscript and file is uploaded in the system.

Attachment Submitted filename: Response to Reviewers.docx

# Editor report: Decision Letter 2

**DOI:** 10.1371/journal.pone.0356646.r005

**Authors:** Salem Salem

6 Aug 2026

Phytochemical Profiling-Guided Green Synthesis and Characterisation of Silver Nanoparticles Using Catunaregam spinosa for Biological Applications

PONE-D-25-68852R2

Dear Dr. Sharma,

We’re pleased to inform you that your manuscript has been judged scientifically suitable for publication and will be formally accepted for publication once it meets all outstanding technical requirements.

Within one week, you’ll receive an e-mail detailing the required amendments. When these have been addressed, you’ll receive a formal acceptance letter and your manuscript will be scheduled for publication.

An invoice will be generated when your article is formally accepted. Please note, if your institution has a publishing partnership with PLOS and your article meets the relevant criteria, all or part of your publication costs will be covered. Please make sure your user information is up-to-date by logging into Editorial Manager at Editorial Manager® and clicking the ‘Update My Information' link at the top of the page. For questions related to billing, please contact billing support.

If your institution or institutions have a press office, please notify them about your upcoming paper to help maximize its impact. If they’ll be preparing press materials, please inform our press team as soon as possible -- no later than 48 hours after receiving the formal acceptance. Your manuscript will remain under strict press embargo until 2 pm Eastern Time on the date of publication. For more information, please contact onepress@plos.org.

Kind regards,

Salem S. Salem

Academic Editor

PLOS One

Additional Editor Comments (optional):

Reviewers' comments:

# Editor report: Acceptance letter

**DOI:** 10.1371/journal.pone.0356646.r006

**Authors:** Salem Salem

PONE-D-25-68852R2

PLOS One

Dear Dr. Sharma,

I'm pleased to inform you that your manuscript has been deemed suitable for publication in PLOS One. Congratulations! Your manuscript is now being handed over to our production team.

At this stage, our production department will prepare your paper for publication. This includes ensuring the following:

* All references, tables, and figures are properly cited

* All relevant supporting information is included in the manuscript submission,

* There are no issues that prevent the paper from being properly typeset

You will receive further instructions from the production team, including instructions on how to review your proof when it is ready. Please keep in mind that we are working through a large volume of accepted articles, so please give us a few days to review your paper and let you know the next and final steps.

Lastly, if your institution or institutions have a press office, please let them know about your upcoming paper now to help maximize its impact. If they'll be preparing press materials, please inform our press team within the next 48 hours. Your manuscript will remain under strict press embargo until 2 pm Eastern Time on the date of publication. For more information, please contact onepress@plos.org.

You will receive an invoice from PLOS for your publication fee after your manuscript has reached the completed accept phase. If you receive an email requesting payment before acceptance or for any other service, this may be a phishing scheme. Learn how to identify phishing emails and protect your accounts at https://explore.plos.org/phishing.

If we can help with anything else, please email us at customercare@plos.org.

Thank you for submitting your work to PLOS One and supporting open access.

Kind regards,

PLOS One Editorial Office Staff

on behalf of

Dr. Salem S. Salem

Academic Editor

PLOS One
